# Multidimensional on-site perception study of stairway spaces in mountain city parks among young and older adult people: a case study of Pipa Mountain Park, Chongqing, China

**DOI:** 10.3389/fpsyg.2025.1620884

**Published:** 2025-08-07

**Authors:** Cong Gong, Xinyu Yang, Changjuan Hu, Zhenkun Mao

**Affiliations:** ^1^Faculty of Architecture and Urban Planning, Chongqing University, Chongqing, China; ^2^Key Laboratory of New Technology for Construction of Cities in Mountain Area, Ministry of Education, Chongqing University, Chongqing, China

**Keywords:** mountain city park, stairway space, on-site study, psychological perception indicator, physiological perception indicator, differences among young and older adults

## Abstract

Stairway spaces in mountain city parks are crucial for vertical traffic. The multidimensional perceptions of stairway spaces are influenced by visual and auditory environments and behavioural traits. The intrinsic associations between environmental factors and age have been universally confirmed using multimodal data; however, localised studies on the perceptions of different age groups in mountainous environments are lacking. Thus, a pilot study was conducted in a mountain city park stairway space in Yuzhong District, Chongqing, China. Controlled environmental variables and real-time measurements of the psychological and physiological perceptions of young and older adult individuals were analysed using summary statistical descriptions of physiological data, Spearman’s correlation analysis for consistency assessment, and a generalised linear mixed model. The influence of eight visual and three auditory environmental factors on physiological perceptions at various nodes along the paths was assessed. Results revealed significant psychological differences between young and older adult individuals on uphill stairway paths. Young participants exhibited higher consistency between psychological and physiological perceptions and more positive psychological responses. Both groups perceived greater stress on long stairway paths than on short ones. The elevation difference, green slope ratio, and openness majorly affected the physiological changes in both groups. These results can guide future stairway space enhancements.

## 1 Introduction

Parks are essential components of the green infrastructure in mountainous cities (Chen et al., 2021). Stairways are critical for vertical traffic flows, provide three-dimensional views, and showcase mountainous terrains (Cheng et al., 2019). Unlike those in flatland cities, mountain parks are closely integrated with undulating topography, and their spatial environments are more complex and dynamic. As a result, users’ perceptual experiences depend on the multisensory coupling of environmental stimuli and movement. However, in current design practice, the landscape differences between mountain and flatland paths are often overlooked. Combined with the physical exertion of ascending and descending in such terrain, this can induce negative emotional states such as anxiety and irritability during use. Thus, accurately measuring the psychological, physiological, and behavioural responses of users in stairway spaces and identifying the most influential landscape factors are essential for enhancing the overall quality of stairway spaces.

In mountain city parks, stairway spaces form unique spatial landscapes and function as crucial traffic networks that connect the upper and lower platforms (Chen et al., 2022). There is an increasing awareness of the need to understand the perception mechanisms of stairway spaces. However, previous studies on mountain city parks have primarily focused on environmental optimisation (Liu et al., 2023), ecosystem service value (Pu et al., 2023), and landscape patterns (Ding et al., 2022). Although studies on stairway spaces have explored environmental preferences (Wang et al., 2022) and perceptual experiences (Gobster and Westphal, 2004), most of the existing research has focused on stairway spaces in plain cities. However, in mountain cities, stairway spaces can provide participants with a significantly different perceptual experience. For example, Li and Liu (2000) showed that body posture and energy consumption significantly differ during uphill and downhill movement in stairway spaces. Nevertheless, the exploration of perceptual experiences in mountain city stairway spaces remains relatively underdeveloped.

The attention restoration theory posits that environments that are attractive, compatible, and moderately complex facilitate the attainment of minimal directional and conscious attention (Kaplan and Kaplan, 1989). According to the stress recovery theory, visiting sensory pleasant surroundings can positively impact stress recovery. This theory not only focuses on the restoration of attention but also includes the recovery of both psychological and physiological perception. The restorative effects are primarily achieved through two mechanisms: (1) psychological mechanisms: manifested as the generation of positive or pleasurable emotions; (2) physiological mechanisms: manifested as the regulation of the autonomic nervous system, including decreases in heart rate (HR) and respiratory rate as well as alterations in electroencephalographic (EEG) activity (Ulrich, 1984; Ulrich et al., 1991). It has been shown that the visual and auditory environments of urban parks significantly influence the psychological perceptions of visitors (Zhang J. et al., 2024a; Zhang R. et al., 2024b). The proportions of sky, plants, and pathways in the visual environment (Cai et al., 2023), as well as other factors such as birdsong, flowing water, and artificial noises in the auditory environment (Erfanian et al., 2021), can influence visitors’ stress perceptions and emotional responses in urban parks. In addition, the interplay of visual and auditory elements in urban green spaces can positively impact visitors’ sensory perception (Deng et al., 2020), with multisensory environments providing significantly improved restorative effects compared to single visual environments. Recent studies have used physiological indicators such as HR variability (Liu et al., 2022), mean arterial pressure (Yin et al., 2023), and respiratory rate (RESP; Li and Kang, 2019). Brainwave indicators such as *α*-EEG, *β*-EEG (Jeon et al., 2023), and β/α (Kislov et al., 2022) and eye movement indicators such as average pupil diameter (APD), saccade frequency (SF), and average fixation time (Liu et al., 2019) were also used to intuitively reflect visitors’ physiological responses of the landscape. Furthermore, related research on urban parks has consistently demonstrated that natural sounds can effectively activate the human parasympathetic nervous system and alleviate stress (Annerstedt et al., 2013).

To assess psychological perceptions, previous studies have used psychological scales such as the profile of mood states (Zeng et al., 2023) and the perceived restorativeness scale (Huang et al., 2023) as well as psychological perception indicators such as visual aesthetic quality and tranquillity rating (Liu et al., 2019) to reflect visitors’ perceptions of the landscape. Recent research on parks has revealed that natural elements can significantly enhance psychological recovery and improve emotional regulation. For example, a higher vegetation coverage rate in parks can promote stress relief (Zeng et al., 2023), while greater openness and natural sounds, such as birdsong and flowing water, can reduce visitors’ mental load (Liu et al., 2019). The direct feedback of individuals to external environmental stimuli can typically be divided into two dimensions: psychological and physiological. However, the relationship between these two dimensions is a complex interactive process. When psychological perception generates a positive response, factors such as the complexity of environmental stimuli, individual differences, and cognitive biases may cause physiological perception to produce a negative response. Therefore, examining the consistency between psychological and physiological perception can better reveal the human–environment interaction. However, related research in this area remains limited. In addition, in mountain city parks, the organization of vertical traffic and the surrounding mountainous landscape cause visitors’ behavioural patterns to change depending on the spatial context (Landais et al., 2020). In stairway spaces, uphill and downhill movements become important factors influencing perception. Researchers have examined the strong correlation between behaviour and urban park perception from various angles, including visitors’ behavioural habits (Jo and Jeon, 2020), activity types (Chen et al., 2024), visit frequency (Ma et al., 2023), and modes of exercise (Lai et al., 2024). However, research on the relationship between perception and behaviour in mountainous environments is lacking.

Previous studies have been conducted in laboratory environments (Dupont et al., 2015) or virtual reality settings (Shi et al., 2020), and interviews and questionnaires have been used to collect perception data from participants (Xie et al., 2024). However, these experimental methods are limited in mountainous city environments. Presentations using photos or videos do not allow participants to authentically perceive the reality of complex terrain and landscape environments. In addition, they cannot fully account for multidimensional perception changes influenced by diverse factors such as the duration of the experience and behavioural activities of visitors. In contrast, using controlled environmental variables, on-site studies can be used to investigate the intrinsic reasons for perception changes in the stairway spaces of mountain parks based on visitors’ on-site experiences and real-time perception feedback. This approach improves the accuracy of measuring visitors’ perceptions of mountainous environments.

Research on population differences and park perception associations by Ode Sang et al. (2016) showed that different genders and age groups exhibit variations in landscape and behaviour preferences. Specifically, the young and older adults display increasingly significant differences in their preferences due to differences in physical function, interests, and recreational pursuits. Ruotolo et al. (2024) explored whether age affects the perception of visual and auditory environments in urban parks and indicated that young participants gave more importance to the evaluation of visual-only elements, while older adult participants showed the opposite preference. It has been shown that sensory function declines owing to the effects of ageing on leisure activity patterns, physical and mental health, and environmental perceptions and experiences (Chang et al., 2020). Additionally, the need for social health and wellness among the older adults influences their perception and evaluation of park spaces (Qiu et al., 2021; Lin et al., 2024). However, perceptions of the young are largely influenced by environmental features and activities (Dai et al., 2022). This is because their generally good health and desire for recreational socialising drive their preference for more dynamic and diverse spatial experiences in park environments (Taczanowska et al., 2024). Although current research has largely revealed the relationship between populations and perceptions, in mountain city parks, the topographical and altitudinal heterogeneities that characterise such parks could mean that perception is influenced not only by spatial characteristics but also by traits inherent in the people that use the parks. Therefore, this study incorporated modes of movement into the analysis to explore perception from a multidimensional perspective.

In summary, traditional research methods to elucidate age-related differences in park perception preferences often rely on unimodal physiological data, evaluation scales, semi-structured interviews, and behavioural annotations (Zhang et al., 2023). This approach is usually impacted by a certain degree of subjectivity and singularity. However, technological innovations and the diversification of data types have enabled multidimensional data coupling analyses in areas such as restorative studies in parks or green spaces (Jeon et al., 2023), soundscape perception (Li and Kang, 2019), and landscape preferences (Ren, 2019). These research methods effectively complement information and help to comprehensively elucidate the relationships between behaviour, space, and perception. As previously mentioned, extensive research has been conducted on the impacts of various visual and auditory environments and behavioural characteristics on the individuals’ perceptions in parks. However, research regarding on-site perceptions in mountain city parks and stairway spaces remains to be conducted, especially with age differences taken into account. To address this gap in knowledge, we based our study on the stress recovery theory and attention restoration theory. Specifically, we aimed to use them to explore the impact of visual and auditory environments as well as modes of movement on the embodied perceptions of young and older adult individuals in stairway spaces within mountain parks from a multidimensional perception perspective. The study utilised wearable ergonomics equipment, supplemented by an environmental satisfaction questionnaire, to conduct experiments in a real-world setting. Two stairway spaces of different lengths were selected, forming four contrasting paths through the combination of uphill and downhill movements as the spatial study objects. Nodes were established at the starting and ending points of the paths, as well as at turning points and platforms, to capture changes in behaviour, time, and visual–auditory environments. Young and older adult individuals were chosen as comparative study groups, aiming to explore the perceptual differences between young and older adult individuals in stairway spaces and the specific factors influencing their perceptions. Overall, we aimed to address the following questions:

1. Do the psychological indicators of young and older adult individuals differ across the four path types? Do physiological indicators change at various nodes along the four paths as the experiment progresses, and what types of changes occur?
2. Do the “psychological-physiological” perceptions of young and older adult individuals show consistent trends across different path types?
3. Do the physiological indicators of young and older adult individuals differ according to path type?
4. How do the visual and auditory environmental characteristics of different spatial nodes affect the physiological indicators of young and older adult individuals?

Our hypotheses were as follows:

*H1*: Due to differences in activity needs and landscape preferences between young and older adult individuals (Ode Sang et al., 2016), their psychological perceptions at each node will differ. Additionally, during the experiment along the four paths, the physiological indicators of young and older adult individuals will show different trends of change between the nodes. Because of the higher responsiveness of the sympathetic nervous system in young individuals (Mcardle et al., 2015), the changes in their physiological indicators during the uphill process will be more pronounced than those in older adult individuals.

*H2*: The psychological perceptions and physiological responses of young and older adult individuals will show inconsistent trends of change along the four paths, with the inconsistency in older adult individuals being greater than that in young individuals. As age increases, the efficiency of neural signal transmission decreases. When exposed to environmental stimuli, the brain and various organs are unable to quickly and accurately process sensory information from different parts of the body and immediately respond with corresponding physiological reactions, leading to inconsistencies in psychological perceptions and physiological responses (Chang et al., 2020).

*H3*: If differences in the perception of various environments exist due to the different physical conditions and activity preferences of young and older adult individuals, the variation in terrain in mountain city parks will affect the visual and auditory stimuli experienced by participants along the four paths. Moreover, the differences in age and movement style will influence participants’ postures and physical activities (Landais et al., 2020), leading to variations in their physiological responses and stress perception along the four paths. The longer and steeper paths with greater elevation difference will result in higher physical exertion, which, compared to shorter paths, will create a greater stress perception for both young and older adult individuals.

*H4*: In mountain city parks, elevation difference and natural elements will be the primary factors influencing the perceptions of young and older adult individuals. Due to factors such as the risk of falls and higher muscle load (Landais et al., 2020), the negative impact of elevation difference on stress perception is greater for older adult individuals than for young individuals. However, higher levels of natural sound or canopy cover can somewhat alleviate the negative emotions of older adult individuals. The specific impacts of visual and auditory environmental characteristics on the physiological indicators of young and older adult individuals will be examined using a generalised linear mixed model (GLMM), with the environmental characteristics of each path as independent variables and physiological, EEG, and eye-tracking data as dependent variables, and the results will be analysed accordingly.

**Figure 1:**
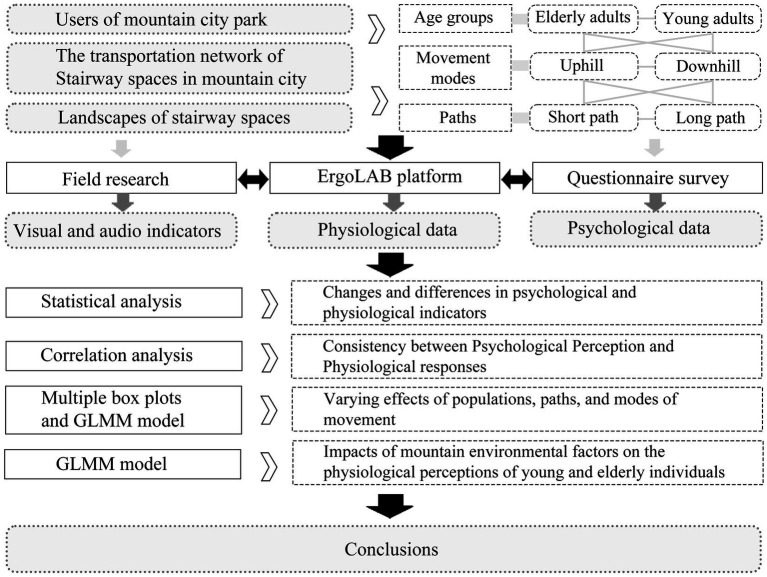
Research framework.

The research framework and analysis procedure are presented in Figure 1.

## 2 Materials and methods

### 2.1 Study area

Pipa Mountain Park in Yuzhong District, Chongqing, southwest China, was selected as the study area. The park has an elevation of 345 m with a height difference of approximately 70 m. It is the highest point in Chongqing’s old city and is one of the highest points in Yuzhong District. It has two main entrances adjacent to primary and secondary urban roads. Pipa Mountain Park has a unique terraced spatial organisation, making it a representative mountain stair space. The park’s winding mountain paths and stable annual temperature range from 15°C to 23°C, with minimal temperature and humidity fluctuations, effectively minimise the impact of other environmental variables on the experiment, providing an ideal setting for ergonomic studies.

**Figure 2:**
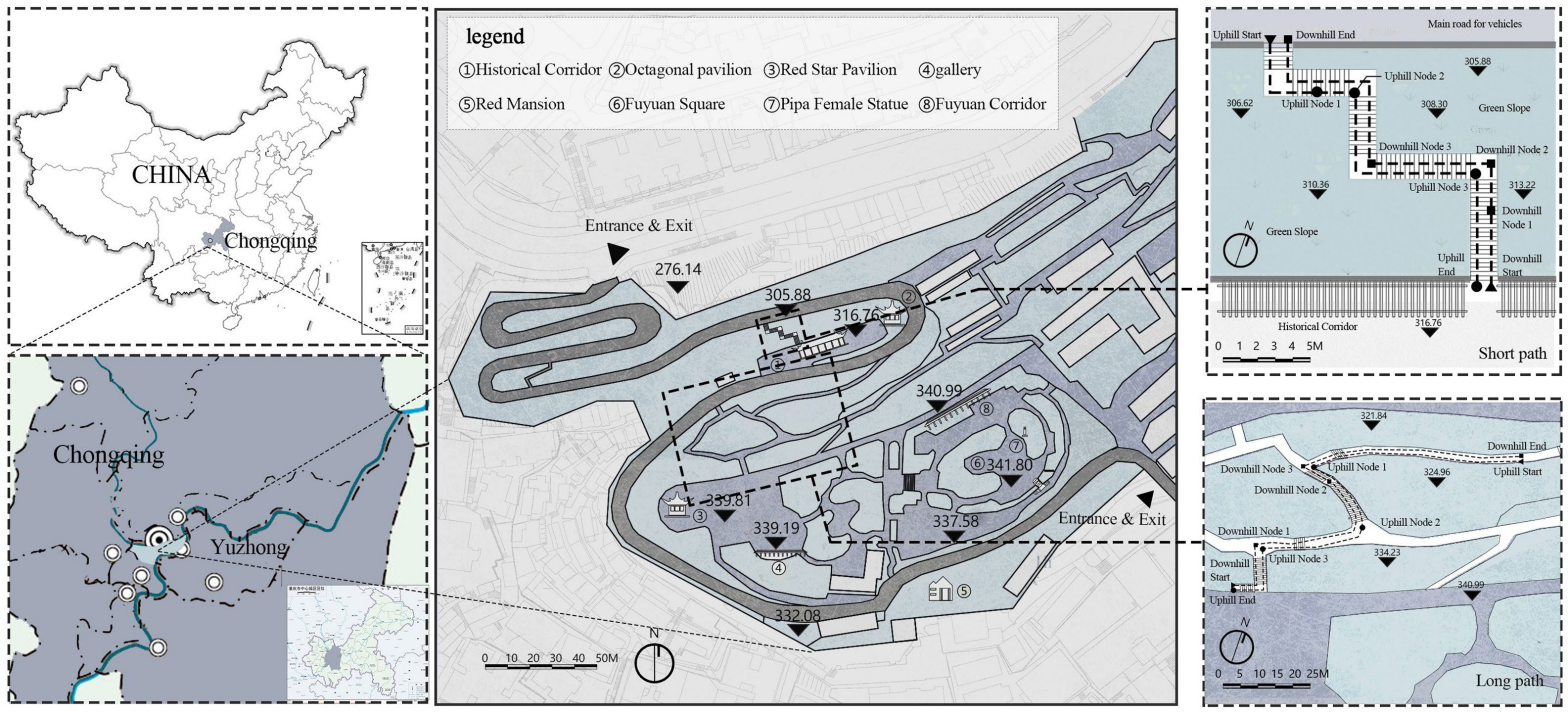
Site overview.

**Figure 3:**
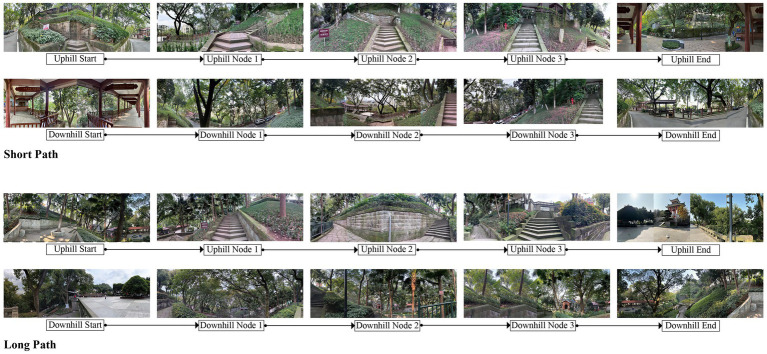
Panoramic views of each nodes.

Two stairways within the park were selected as spatial objects to explore ergonomic differences between young and older adult individuals (Figure 1). Both stairways use right-angle plains to connect the upper and lower spaces of the park. They are paved with artificial stone slabs and are rich in surrounding elements such as plants, retaining walls, green slopes, and railings. Both stairways have four turns, but their lengths differ. Basic lighting fixtures are also provided along the stairways. Specifically, the long stairway has 152 steps with a total path length of 45.6 m and a height difference of 13.26 m between the start and end points. In contrast, the short stairway has 79 steps with a total path length of 23.7 m and a height difference of 10.88 m. To account for differences in walking behaviour, the experiment included both uphill and downhill stairway roaming tests. Based on a preliminary experiment, four paths were established as spatial research subjects: long path uphill (LPU), long path downhill (LPD), short path uphill (SPU), and short path downhill (SPD). Five key perception nodes were set along each of the four paths (Figures 2, 3).

### 2.2 Participants and study design

Fifty-two participants were recruited for this study who had no history of cardiovascular disease, mental illness, or neurological disorder, did not regularly take prescription drugs, had uncorrected or corrected vision below 1.0, and had good sensory abilities (primarily vision and hearing). Among them, 27 were classified as young individuals (18–44 years) (M = 20, SD = 3.07) and 25 were classified as older adults (60–74 years) (M = 68, SD = 3.98), with an approximate gender ratio of 1:1. Participants were required to not consume stimulative beverages for 1 day prior to the experiment, to ensure adequate sleep and good physical condition. All participants voluntarily enrolled in the experiment; they were fully informed about and agreed to all procedures. Additionally, the participants consented to the use of their data for research purposes. After checking data integrity and conducting preliminary analysis to screen out anomalies, 48 valid samples were obtained, including 25 young individuals (12 males, 13 females) and 23 older adult individuals (11 males, 12 females). Specifically, data entries with missing values, abnormal sensor outputs, or extreme outliers exceeding ±3 standard deviations from the group mean were excluded to ensure validity and reliability of the dataset.

For the two uphill and downhill stairway paths, the research team set up fixed walking routes with stopping points established at the start and end points of the stairways, at turns, and on the platforms. Each participant was required to walk at a constant speed and stay at each stopping point for approximately 15 s. While going uphill, participants were instructed to look straight ahead or upward at an angle of 0°-45°, and while going downhill, to look straight ahead or downward at an angle of 0°-45°, with their field of view controlled between 120° and 180°. The experiment set up three groups of independent variables: “path length” (long or short), “movement direction” (uphill or downhill), and “age group” (older adult or young individuals). The visual and auditory environment indicators at each stopping point along the four paths were measured, in addition to the overall visual and auditory psychological perception indicators and physiological (perceptual) indicators such as electrodermal activity, brainwaves, and eye movements of the participants at each stopping point. In addition, the experiment strictly controlled weather conditions such as temperature and humidity (temperature was 18–23°C, humidity between 60 and 80%) and lighting (controlled between 500 and 2000 lux). A Lotoo PAW-VE high-fidelity recorder was used for audio acquisition, and the recordings were normalised using an Adobe Audition digital audio workstation.

### 2.3 Measurements

The perceptual differences of the participants on different paths were assessed using heart rate variability (HRV), RESP, EEG, and eye-tracking as indicators (Table 1). HRV is associated with the interactions between the sympathetic and parasympathetic nervous systems and can objectively and reliably assess the activity of the autonomic nervous system, reflecting changes in participants’ psychological states (Pham et al., 2021). Therefore, two main parameters were selected for HRV, defined as follows: 1. HR (unit: bpm) is the mean number of heart beats per minute; 2. Low-frequency to high-frequency power ratio (LF/HF; unit: ms^2^/Hz) is the ratio of the power of the low-frequency component to the high-frequency component of the HRV. The RESP was defined as the number of breaths taken per minute. This parameter is frequently measured alongside HRV to provide a comprehensive view of autonomic functions and stress responses (Brown and Gerbarg, 2005). An EEG is a valuable tool for assessing emotional and stress-related changes because it records electrical activity in the brain (Bazanova and Vernon, 2014). The present study used EEG-*α* (8–13 Hz), EEG-*β* (14–30 Hz), and β/α, which are EEG measurements, because they best reflect emotional and stress changes. Eye-tracking metrics are valuable indicators of cognitive processes, including attention and stress. This study selected APD, fixation frequency, and SF as the primary metrics for measuring participants’ cognitive load, emotional state, and attention. The experimental procedure was programmed using the ErgoLab platform (Kingfar International Inc., Beijing, China). The acquisition of physiological and EEG data required participants to wear the ErgoLAB chest smart wearable sensor (i.e., which was attached to the left chest), finger smart wearable sensor, ear smart wearable sensor (i.e., clipped to the centre of the earlobe), and water-electrode EEG system. After properly wearing the electrode cap, participants soaked the sponges in saline solution and placed them into the electrode slots, positioning the electrodes according to the standard electrode placement guidelines. These sensors were connected to the laptop via Bluetooth 5.0 to transmit real-time data to the ErgoLAB platform. Eye-tracking calibration and the start/stop of data recording were managed using the Tobii Pro Glasses Controller software on the same laptop.

**Table 1:** Physiological signals, EEG, and eye movement-related metrics and explanations.

Hierarchy	Indicator	Description	Equipment
Psychological	Heart rate	Heart rate is increased with elevated activity levels, stressful conditions, and stimulation but decreased by quiet and relaxed states (Pham et al., 2021).	ErgoLAB Finger Biosensing Smart Wearable Finger Sensor ErgoLAB Wrist Biosensing Smart Wearable Wrist Sensor ErgoLAB Chest Biosensing Smart Wearable Chest Strap Sensor
	Respiratory rate	An index of the balance between sympathetic and parasympathetic activity. A high respiratory rate is typically associated with stress, anxiety, and tension. A low respiratory rate is usually associated with relaxation and meditative states (Pham et al., 2021).	
	Low-frequency/high-frequency ratio	A higher ratio suggests dominance of sympathetic activity (stress or arousal), whereas a lower ratio indicates higher parasympathetic activity (relaxation) (Immanuel et al., 2023).	
Electroencephalography	α-EEG	Increased alpha activity is commonly observed when an individual is in a relaxed, meditative state, or during quiet wakefulness with eyes closed (Laufs et al., 2003).	ErgoLAB EEG Wearable Hydrogel Electrode EEG Device
	β-EEG	High beta activity is associated with heightened cognitive activity, attention, stress, and anxiety (Palacios-García et al., 2021).	
	*β*/*α*	A higher β/α ratio often indicates increased stress and anxiety levels, whereas a lower ratio suggests a more relaxed state (Palacios-García et al., 2021).	
Eye-tracking	Average pupil diameter	An increase in pupil size (pupil dilation) is often associated with a higher cognitive load, increased attention, and stress or arousal. Conversely, pupil constriction can indicate a state of relaxation or low cognitive demand (Beatty and Lucero-Wagoner, 2000).	Tobii Pro Glasses2 wearable eye tracker
	Fixation frequency	Higher fixation rates can indicate sustained attention and detailed processing of visual information, whereas lower fixation rates might suggest distraction or a broader, less focused scan of the environment (Holmqvist et al., 2011).	
	Saccade frequency	A higher saccade rate can indicate exploratory behaviour and the search for new information, often associated with increased cognitive load or stress. A lower saccade rate may indicate focused attention and fewer transitions between visual targets (Holmqvist et al., 2011).	

EEG, electroencephalographic activity.

To collect participants’ perception responses to the stairway spaces, a “Visual and Auditory Perception Evaluation Scale” was constructed, combined with the research objectives, and based on eight visual perception indicators widely used in landscape assessment and design: tranquillity, naturalness, openness, richness in species, refuge (enclosure and safety), prospect, sociality, and cultural significance (Grahn and Stigsdotter, 2010), and the international soundscape research standards (International Organization for Stadardization, 2017). This scale covers multiple dimensions such as refuge, altitude perception, naturalness, diversity, publicness, aesthetics, recognisability, functionality, accessibility, effort and ease, anxiety and calmness, indifference and excitement, pleasantness, annoyance, tranquillity, disorder, vibrancy, monotony, eventfulness, lack of events, naturalness of sound, and diversity of sound. The questionnaire was divided into three categories: participant social information, visual perception evaluation, and auditory perception evaluation.

Visual and auditory environments and their interactions can influence participants’ perceptions at various nodes within the stairway spaces of mountain city parks. To investigate which spatial elements in stairway spaces significantly affected participants’ embodied perceptions, the selected indicators should reflect the mountainous terrain and landscape characteristics and comprehensively cover the stairway components along the experimental paths. Therefore, the final spatial visual and auditory environment indicators for the nodes included “openness, green view index, concealment, material hardness ratio, proportion of retaining walls, proportion of green slopes, proportion of trees, and elevation difference.” The spatial auditory environment indicators included “the proportion of natural, artificial, and mechanical sounds during the stopping time at each node.” In addition, the “elevation difference between the current node and the previous node” was used as an indicator of the relationship between spatial nodes and behaviour. Using on-site surveys, recording, and statistical analysis of the dynamic spatial auditory environment indicators and behaviour-related indicators, spatial photographs were taken at the geometric centre of each node, capturing images in the east, south, west, and north directions according to observational habits to create panoramic images. Photoshop was used to manually delineate the outer boundaries of each visual factor, and the proportion of each segment to the total pixel area was calculated to determine the final values of each spatial element for subsequent analysis (Table 2).

**Table 2:** Spatial indicators in mountainous historic urban parks.

Hierarchy	Category	Description	Calculation method*
Visual	Openness	Percentage of prospect view pixels (PVPixel) of the street view image	$\text{Openness}=\frac{\text{PVPixel}}{P}$
	Green view index	Percentage of greenery pixels (GreeneryPixel) of the street view image	$\text{Green view index}=\frac{\text{GreeneryPixel}}{P}\;$
	Concealment	Percentage of obstructed pixels (ConcealmentPixel) in the street view image	$\text{Concealment}=\frac{\text{ConcealmentPixel}}{P}$
	Material hardness ratio	Ratio of the percentage of soft material pixels (SoftMaterialPixel) to the percentage of hard material pixels (HardMaterialPixel) in the street view image	$\text{Material hardness ratio}=(\frac{\text{SoftmaterialPixel}}{\text{HardMaterialPixel}})\times 100\%$
	Retaining walls ratio	Percentage of retaining walls pixels (RetainingwallsPixel) in the street view image	$\text{Retaining walls ratio}=\frac{\text{RetainingwallsPixel}}{P}$
	Green slopes ratio	Percentage of retaining walls pixels (GreenslopesPixel) in the street view image	$\text{Green slpoes ratio}=\frac{\text{GreenslopesPixel}}{P}$
	Proportion of trees	Percentage of tree canopies pixels (TreesPixel) in the street view image	$\text{Proportion of trees}=\frac{\text{TreesPixel}}{P}$
	Elevation difference	The elevation drop gradient near the adjacent node.	$\text{Elevation difference}={\mathrm{Ele}}_{i}-\mathrm{El}e_{\mathrm{ni}}$
Acoustic	Natural sound	Average percentage of naturalsound ($T_{\mathrm{ns}}$) duration in the recorded segment	$\text{Natural sound}=\frac{1}{n}\sum_{i=1}^{n}\frac{T_{\mathrm{ws},i}}{T_{\text{total},i}}$
	Artificial sound	Average percentage of artificial sound ($T_{\mathrm{as}}$) duration in the recorded segment	$\text{Artificial sound}=\frac{1}{n}\sum_{i=1}^{n}\frac{T_{\mathrm{ws},i}}{T_{\text{total},i}}$
	Mechanic sound	Average percentage of mechanic sound duration ($T_{\mathrm{ms}}$) in the recorded segment	$\text{Mechanic sound}=\frac{1}{n}\sum_{i=1}^{n}\frac{T_{\mathrm{bs},i}}{T_{\text{total},i}}$

*P is the total pixels of the street view image, “*T_total_*” is the total number of recorded segments.

### 2.4 Experimental procedure

The experiment was conducted over 2 weeks, from November 6, 2023, to November 19, 2023. Before the experiment, the research team provided participants with a 15-min video training session that included information on experimental paths, equipment usage, precautions, and confidentiality principles.

**Figure 4:**
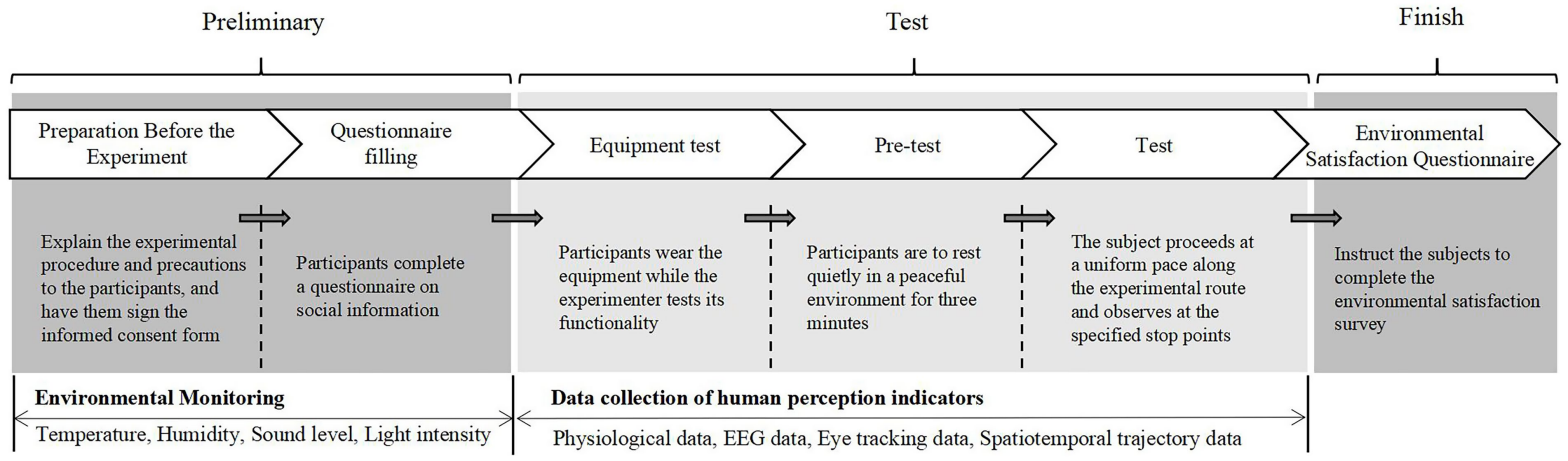
Flow diagram of the experimental procedure.

After helping the participants to correctly wear the experimental equipment, the participants rested for approximately 3 min, followed by calibration of the eye tracker, and synchronisation of the ErgoLAB platform and eye-tracking recordings. Each participant spent approximately 60 min completing the four paths, with each stairway path perception experiment lasting approximately 5–7 min. Before proceeding to the next stairway path experiment, the participants sat and rested for 8–10 min. Participants followed the sequence of “LPD-SPD-SPU-LPU.” They stayed for approximately 15 s at each predetermined path node and observed the surrounding environment from a preset perspective (Figure 4). During the walk, the primary researcher guided the participants along the paths and attempted to maintain a normal walking pace. The ErgoLAB DataLOG APP mobile terminal human factor recording system was used to synchronise the path records. Simultaneously, 2–3 experimenters followed the participants and recorded real-time objective environmental indicators at each path node, such as A-weighted equivalent sound levels, temperature, humidity, and illumination. The entire experiment was recorded. After completing each path, the primary researcher and two experimenters communicated with the participants and helped them fill out the “Visual and Auditory Perception Evaluation Scale.” Each participant performed the experiment twice, and the average value was taken to reduce errors. After the experiment, the physiological data, eye-tracking recordings, and behavioural trajectories were synchronised on the ErgoLAB platform to obtain a complete dataset.

### 2.5 Statistical analyses

After the on-site ergonomic experiment, the research team conducted an integrity check on the physiological measurements, eye-tracking recordings, and EEG recordings. This integrity check was conducted by synchronising the eye tracker and physiological datasets. Specifically, all the data were integrated and matched on the ErgoLAB platform. The perception questionnaires were organised and entered into the “Questionnaire Star” app for data entry and integrated statistics. Segment analysis was then conducted on the synchronised recordings of the experiment, with 48 datasets selected.

Using the ErgoLAB platform, the physiological, EEG, and eye-tracking data were pre-processed using the corresponding data analysis modules. First, the experimental data were segmented at predefined nodes. Data were then extracted from the 48 experiments at five nodes and four path segments, resulting in a total of 4,752 node segments. The physiological data were filtered and corrected for anomalies using the filtering and anomaly correction functions in the ErgoLAB data analysis module. HR, RESP, and LF/HF data for each segment were then extracted according to the node segment divisions. EEG data were filtered using the ErgoLAB platform with high- and low-pass filters set to 0.1 and 30 Hz, respectively. After removing the filtering artefacts, the *α*-EEG, *β*-EEG, and β/α data for each node segment were exported. Eye-tracking data were then processed in the eye-tracking module of the ErgoLAB platform, where I-VT filters were used to filter noise and classify saccades and fixation samples. Sliding mean and median filters were applied for denoising, and the pupil diameter, fixation count, and saccade count were then exported. After preprocessing the physiological data on the platform, normalisation was used to eliminate individual differences in the physiological signals due to the varying units, dimensions, and magnitudes of the measurement indicators (Singh and Singh, 2020), allowing for standardised and multidimensional data coupling analysis.

R version 4.4.1 was used for data analysis. The data were analysed using the following methods: (1) Descriptive statistics of the psychological and physiological data were obtained using dumbbell and line charts to visually reflect the differences in perception evaluations and physiological change trends during the traversal process. (2) Spearman correlation analysis was performed on the psychological and physiological data to explore the consistency between psychological and physiological indicators. (3) Multiple box plots were used to preliminarily compare the physiological data differences. As the dependent variables did not meet the assumption of normality based on preliminary diagnostics, a generalised linear mixed model (GLMM) with appropriate distribution assumptions was applied. In this model, age group, path, movement mode, and their interaction terms were treated as fixed effects, and participant ID was included as a random effect to control for inter-individual variability. Age group, path, and movement mode were set as independent variables, while the physiological, EEG, and eye-tracking indicators collected during the experiment were used as dependent variables. This model was used to explore the effects of age group, path, movement mode, and their interactions on the perceptions of young and older adult individuals. Additionally, the stress perception levels and emotional arousal of the young and older adult individuals along the four stairway paths were ranked. (4) Furthermore, an GLMM was applied, with spatial characteristics of each path as independent variables and physiological, EEG, and eye-tracking indicators as dependent variables; these steps were conducted to explore the impact of spatial features and sound environment characteristics on physiological indicators.

## 3 Results

The results below are mainly divided into four parts: (1) the trends and differences in the psychological and physiological data of the young and older adult groups across the four pathways (Section 3.1); (2) the consistency in psychological and physiological perception trends of the young and older adults across the four paths (Section 3.2); (3) the differences in physiological indicators of the young and older adult groups across the four pathways and ranking of perceived stress levels across the four pathways (Section 3.3); and (4) the impact of visual and auditory environmental characteristics on physiological indicators of the young and older adults across the four paths (Section 3.4).

### 3.1 Statistical analysis of the psychological and physiological indicators

#### 3.1.1 Statistical analysis of the psychological indicators

**Figure 5:**
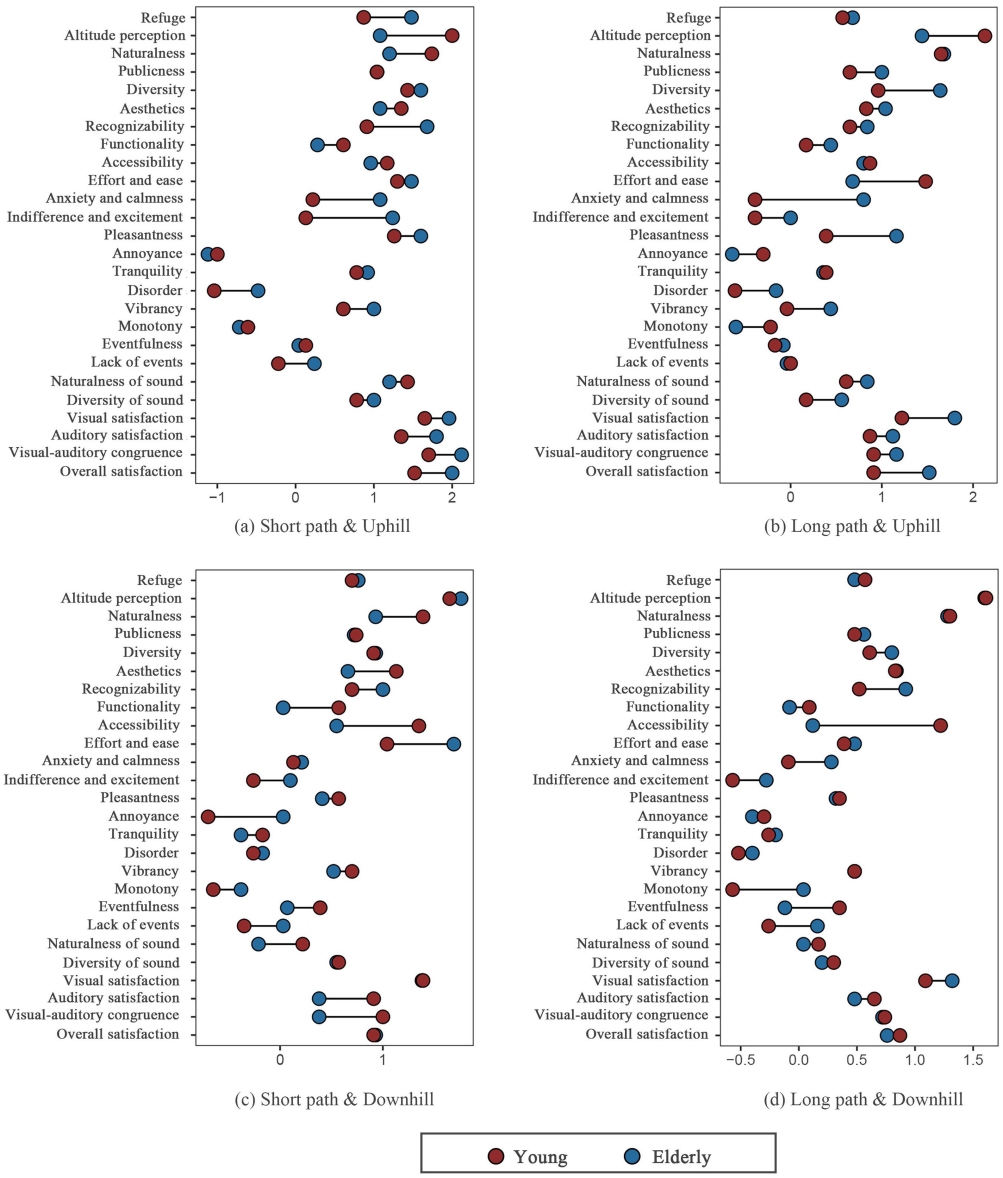
Psychological responses of the young and older adults across different paths.

A total of 48 questionnaires were collected, and their reliability and validity were tested (KMO > 0.6; Bartlett’s test of sphericity significance *p* < 0.001). Twenty-six psychological perception indicators were then analysed. Participants’ overall satisfaction, visual and auditory satisfaction, and visual–auditory congruence were significantly higher for the long paths than for the short paths, with overall satisfaction being slightly higher for the uphill paths than for the downhill paths. (1) For the LPU, there were apparent differences in the visual indicator “Recognisability” and the auditory indicator “Exciting” between young and older adult individuals (Figure 5). Young individuals perceived the LPU as more recognisable and found the sound environment exciting. (2) For the LPD, clear differences were observed in the auditory indicator “Pleasantness,” with older adult individuals finding the sound environment more chaotic and unpleasant. (3) For the SPU, noticeable perceptual differences emerged in the visual indicator “Refuge” and the auditory indicator “Annoying,” with young individuals feeling that the refuge was poorer and the sound environment was more unsettling and irritating. (4) For the SPD, young and older adult individuals showed apparent differences in the visual indicator of “Accessibility.” Compared to older adult individuals, young individuals had a better perception of “Accessibility.” These results highlight significant differences in the perceptions of young and older adult individuals across the four paths, with the uphill path differences being more pronounced.

#### 3.1.2 Statistical analysis of the physiological indicators

**Figure 6:**
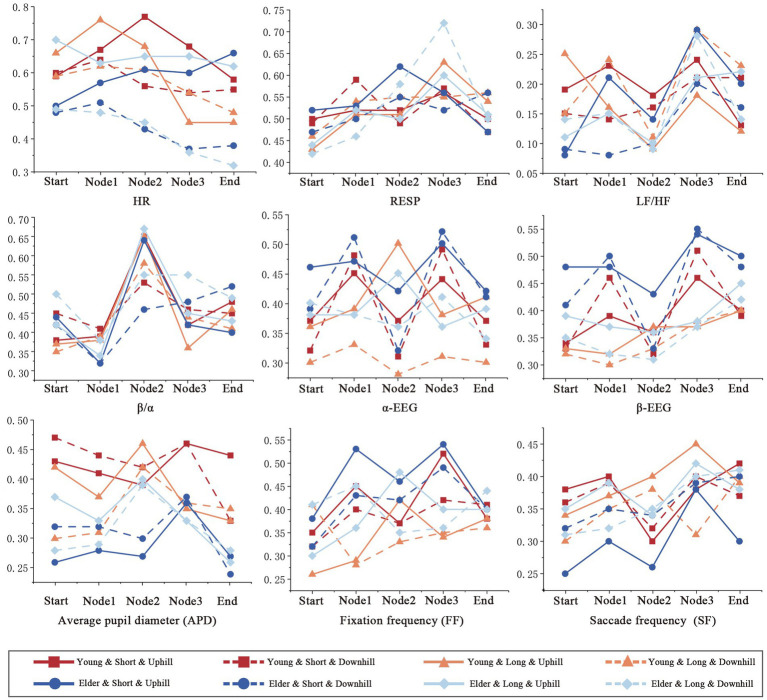
Comparison of physiological indicators among the young and older adults on different paths.

The physiological indicators measured at the start, Node 1, Node 2, Node 3, and end points of the experimental paths were taken as time variables to statistically describe the changes in physiological, EEG, and eye-tracking indicators of young and older adult individuals on the four paths and for further data analysis (Figure 6). (1) The results demonstrate that for the LPU, the HR of young and older adult individuals exhibited opposite trends after Node 2, with the HRs of older adult individuals gradually increasing. The LF/HF trends for both young and older adult individuals were similar, reaching their lowest values at Node 2 and then generally increasing. However, the LF/HF ratio of older adult individuals was considerably higher than that of young individuals after Node 3. The APD of older adult individuals was generally lower than that of young individuals overall, showing a significant increase from Node 2 to Node 3 before decreasing. (2) For LPD, HR was significantly lower in older adults than in young individuals. The *β*/*α* ratios of the young and older adult individuals showed opposite trends after Node 2; they gradually decreased and increased, respectively. (3) For the SPU, the HR of older adult individuals remained relatively stable, whereas the HR of young individuals gradually decreased after Node 1. The LF/HF ratio of older adult individuals showed an overall upward trend, whereas that of young individuals showed a sharp decline from the beginning. The SF of older adult individuals peaked at Node 3 before gradually decreasing, whereas that of young individuals significantly decreased at Node 2 before increasing. For the SPU, the physiological indicators of young and older adult individuals exhibited similar trends. (4) For the SPD, the HR of both young and older adult individuals gradually decreased, but the HR of older adult individuals was lower than that of young individuals. The RESP of older adult individuals remained relatively stable, whereas that of young individuals rose sharply after Node 1 and peaked at Node 3. These results demonstrate that young and older adult individuals exhibit differences in their psychological perceptions and physiological responses on the different path types.

### 3.2 Correlation analysis of psychological and physiological indicators

**Figure 7:**
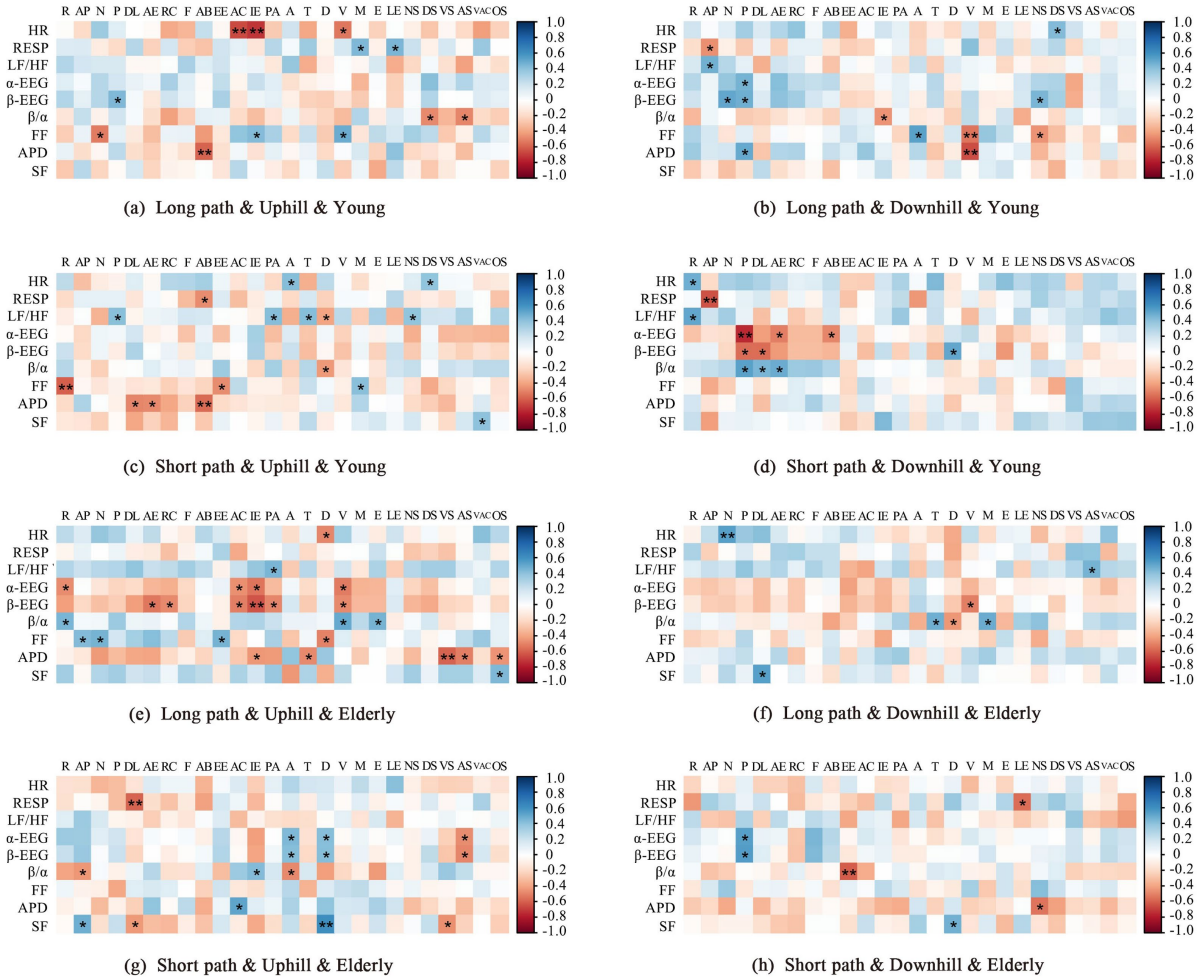
Correlation analysis of psychological and physiological indicators of young and older adult individuals on different paths. *Indicates a significant correlation at the 0.05 level, whereas **indicates one at the 0.01 level. R, refuge; AP, altitude perception; N, naturalness; P, publicness; DL, diversity; AE, aesthetics; RC, recognisability; F, functionality; AB, accessibility; EE, effort and ease; AC, anxiety and calmness; IE, indifference and excitement; PA, pleasantness; A, annoyance; T, tranquillity; D, disorder; V, vibrancy; M, monotony; E, eventfulness; LE, lack of events; NS, naturalness of sound; DS, diversity of sound; VS, visual satisfaction; AS, auditory satisfaction; VAC, visual–auditory congruence; OS, overall satisfaction.

To explore the relationship between the perceptions of participants and objective physiological measurements, a Spearman correlation analysis was performed on the physiological data and perception evaluation questionnaire scores for the four paths (Figure 7). We found that young individuals had more positive psychological and physiological perceptions during the experiment, whereas older adult individuals responded most negatively during high-intensity activities. (1) For young individuals, the HR during the LPU was significantly positively correlated with “anxiety and calmness” and *β*-EEG was negatively correlated with “publicness”; for the LPD, APD was significantly positively correlated with “vibrancy,” whereas HR was negatively correlated with the “diversity of sound” of the auditory environment; for the SPU, LF/HF was negatively correlated with the visual environment’s “publicness” and the auditory environment’s “pleasant” and “tranquil” qualities; and for the SPD, *α*-EEG was significantly positively correlated with “publicness,” and LF/HF was negatively correlated with “refuge.” The above results indicate that the changes in psychological and physiological perceptions of young individuals across the four paths are relatively consistent, and environments with higher publicness can effectively alleviate their stress. (2) For older adult individuals, during the LPU, LF/HF was negatively correlated with “pleasantness,” and β-EEG was positively correlated with “recognisability” and “indifference and excitement”; for the LPD, HR was significantly negatively correlated with “naturalness,” whereas β/α was positively correlated with the “disorder” of the auditory environment; during the SPU, β/α was positively correlated with altitude perception,” and the SF was significantly negatively correlated with the auditory environment’s “disorder.” Additionally, for the SPD, β/α was significantly positively correlated with “effort and ease.” The changes in psychological and physiological perceptions of older adult individuals exhibited some inconsistency, with disordered environments and elevation differences acting as potent stressors. Overall, these findings show that although certain psychological and physiological indicators represent the same emotional perception, young and older adult individuals show some inconsistency between psychological and physiological perceptions of emotions across the four paths; this can largely be attributed to the two groups having inherent differences in perception sensitivity, preferences, etc. This inconsistency is more pronounced in older adult individuals than in young individuals.

### 3.3 Perceptual analysis of four ladder trails

#### 3.3.1 Physiological stress level comparison for four ladder trails

**Figure 8:**
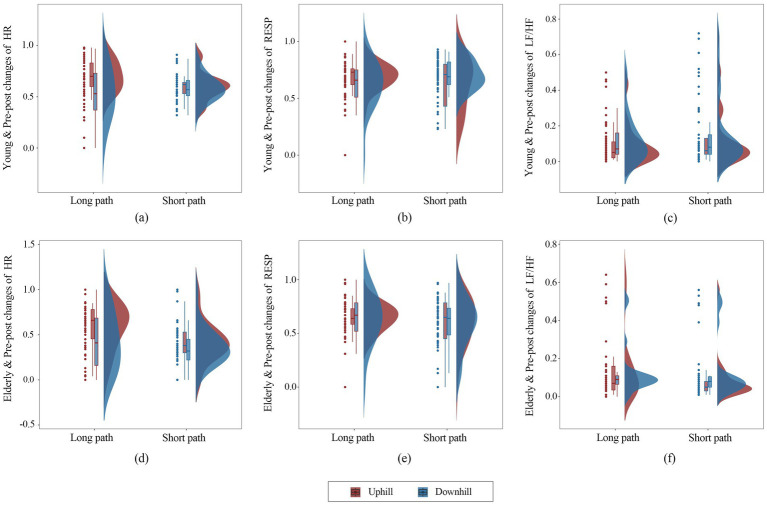
Box plots presenting data for different populations, paths, and modes of movement based on differences in physiological electrical indicators.

Changes in HR, RESP, and LF/HF for the young and older adult individuals were larger on the long paths than on the short paths (Figure 8). The HRs of young and older adult individuals on the long paths were significantly higher than those on the short paths, and the HR changes on the LPU were significantly higher than those on the LPD. On the short paths, the HR changes for the young individuals were relatively similar on the uphill and downhill paths, whereas those of older adult individuals were slightly higher on the downhill paths than on the uphill paths. Changes in RESP among young and older adult individuals on the LPU were slightly higher than those on downhill paths. For the short paths, RESP was slightly higher on uphill paths than on downhill paths, with young individuals showing greater changes than older adult individuals. The LF/HF ratios for young and older adult individuals were relatively similar on both uphill and downhill paths, with LF/HF being slightly higher on the uphill paths than on the downhill paths, for both long and short paths. These results were used to generate a preliminary ranking of stress perception for young and older adult individuals along four paths: long paths > short paths, uphill > downhill.

#### 3.3.2 Interactive effects of physiological indicators on four ladder trails

In this study, age group, path, and mode of movement were the independent variables used to establish the GLMM. The effects of these variables and their interactions in the physiological data were assessed. The results showed that path, mode of movement, and their interactions had a significant impact on RESP (*p* < 0.05; Table 3), with RESP being higher with uphill movement than with downhill movement. The interaction between path and population had a significant impact on LF/HF (*p* < 0.05), whereas this interaction and the second-order interaction had a significant impact on *β*/*α* (*p* < 0.05 and *p* < 0.01, respectively), with a higher LF/HF on the long paths than on the short paths and a higher ratio in young individuals than in older adult individuals. For average fixation frequency, the interaction between mode of movement and age group and the three-way interaction among path, age group, and mode of movement had significant impacts (*p* < 0.05). For APD, the path had a significant main effect (*p* < 0.05), and second- and third-order interactions among path, age group, and movement mode also had significant effects (*p* < 0.05), with the APD on the short path being slightly larger than that on the long path. For the average SF, the mode of movement and the interaction between the path and mode of movement had significant impacts (*p* < 0.05). However, HR, α-EEG, and β-EEG did not exert significant effects. Based on the fixed effect estimates of the two typical physiological indicators representing participants’ stress perception, LF/HF and *β*/*α*, in the four comparison groups for young and older adult individuals, the stress perception rankings for the four paths were determined. For young individuals, the ranking is: LPU > LPD > SPU > SPD, whereas for older adult individuals, the ranking is: LPU > LPD > SPD > SPU.

**Table 3:** Generalised linear mixed model results.

Indicators	Effect	Estimate	Std.	*t*-value	Sig.
Respiratory rate	Path	0.41	0.26	1.56	*
	Motion	0.49	0.26	1.88	*
	Path*Motion	−0.31	0.17	−1.88	*
LF/HF	Path*People	0.25	0.17	1.51	*
β/α-EEG	Path	−0.46	0.31	−1.51	*
	People	−0.66	0.32	−2.13	*
	Path*People	0.29	0.20	1.50	**
Fixation frequency	Motion*People	−0.19	0.13	−1.42	*
	Path*People*Motion	0.19	0.08	2.26	*
Average pupil diameter	Path	0.62	0.24	2.59	*
	Path*Motion	−0.41	0.15	−2.70	*
	Path*People	−0.43	0.16	−2.78	*
	Motion*People	−0.35	0.16	−2.29	*
	Path*People*Motion	0.33	0.10	3.40	*
Saccade frequency	Motion	0.38	0.26	1.48	*
	Path*Motion	−0.26	0.16	−1.59	*

*Indicates a significant correlation at the 0.05 level, **Indicates one at the 0.01 level. Non-significant main effects and interaction effects are not included in the table. LF/HF: ratio of the power of the low-frequency component to the high-frequency component of the heart rate variability; β/α-EEG, ratio of β waves to α waves in electroencephalography.

### 3.4 Analysis of the spatial audiovisual factors

#### 3.4.1 Audio factor

**Figure 9:**
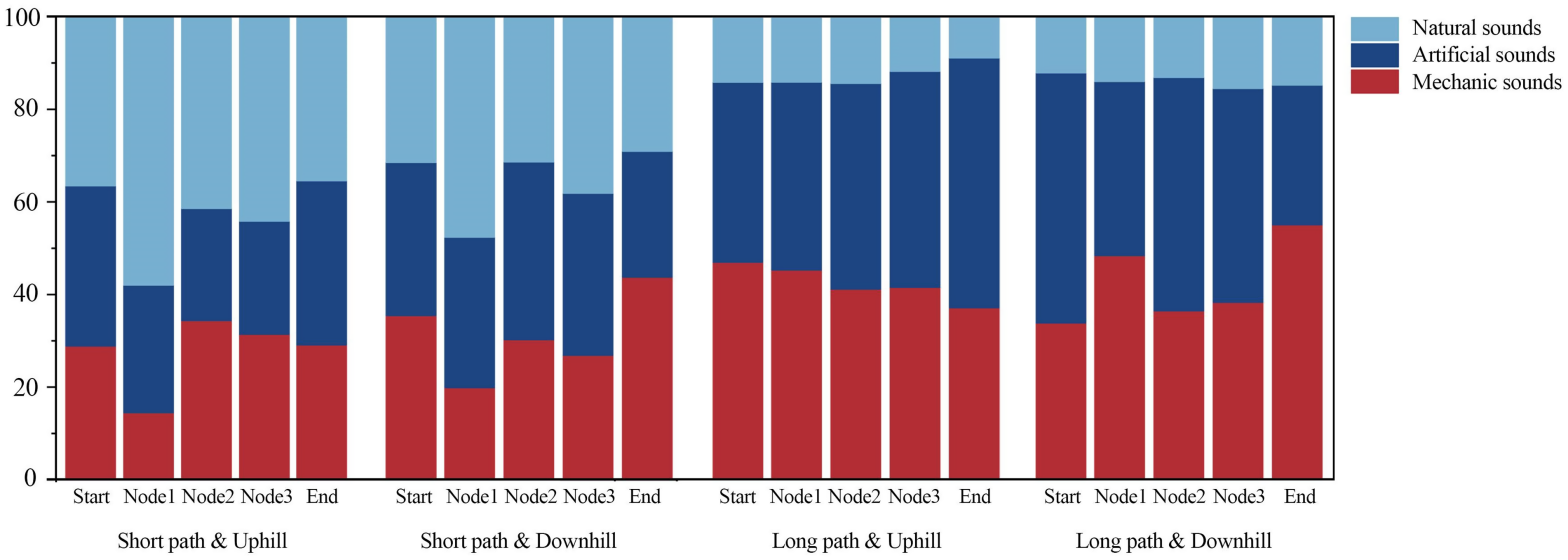
Summary statistics of the acoustic environment.

A statistical analysis of the proportion of various sounds during the stopping time at each node revealed that the sound environment varied significantly depending on the path and movement mode. Compared to the long paths, the short paths had a higher proportion of natural sounds and a lower proportion of artificial sounds. For the SPU and SPD, natural sounds dominated at the start but gradually decreased, and mechanical sounds gradually increased. In the LPU and LPD, the proportion of artificial and natural sounds remained relatively stable across all path nodes, but the proportion of mechanical sounds significantly increased, reaching a peak at the end point (Figure 9).

**Figure 10:**
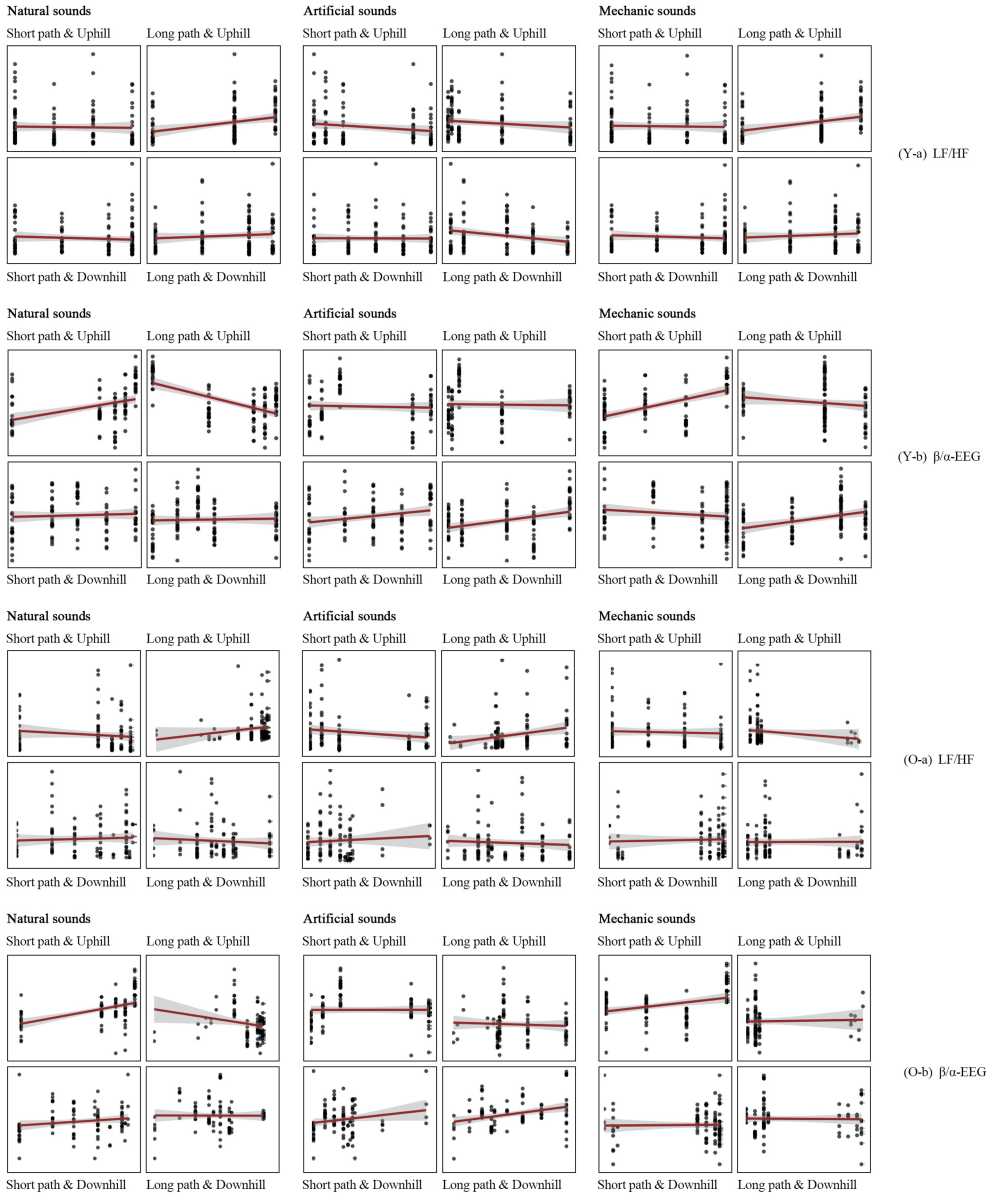
Results of the GLMM for the acoustic environment and physiological indicators of different groups, paths, and modes of movement.

The study used the proportion of natural, artificial, and mechanical sounds at each node as independent variables in the GLMM to analyse changes in the physiological data at different nodes for the different paths and modes of movement. The results indicated significant differences in physiological data due to the sound environments between young and older adult individuals. For young individuals, artificial sound significantly influenced their physiological data, whereas for older adult individuals, this trend was not significant (Supplementary Figure 1). Further path-specific discussions revealed that for the LPU, an increase in mechanical sounds led to a gradual increase in LF/HF among young individuals (Figure 10Y-a). For the LPD and SPD, an increase in artificial sounds caused a gradual increase in *β*/*α* among young individuals (Figure 10). For the SPU, an increase in mechanical sounds also resulted in a gradual increase in β/α among young individuals (Figure 10Y-b). For older adult individuals, artificial and natural sounds had a more significant impact on their physiological data, particularly on *β*/*α* (Supplementary Figure 1). Path-specific discussions indicated that for the LPU, an increase in artificial sounds led to a gradual increase in LF/HF among older adult individuals (Figure 10O-a), whereas an increase in natural sounds resulted in a gradual decrease in *β*/*α* (Figure 10O-b). For the SPU, the *β*/*α* of older adult individuals increased with the rise in mechanical sounds (Figure 10O-b). For the SPD, an increase in artificial sounds led to a gradual increase in LF/HF among older adult individuals (Figure 10O-a). The impact of the sound environment on older adult individuals was not significant on other paths. These findings suggest that the same sound indicators in different path environments may have different physiological perception impacts on young and older adult individuals.

#### 3.4.2 Visual factors

**Figure 11:**
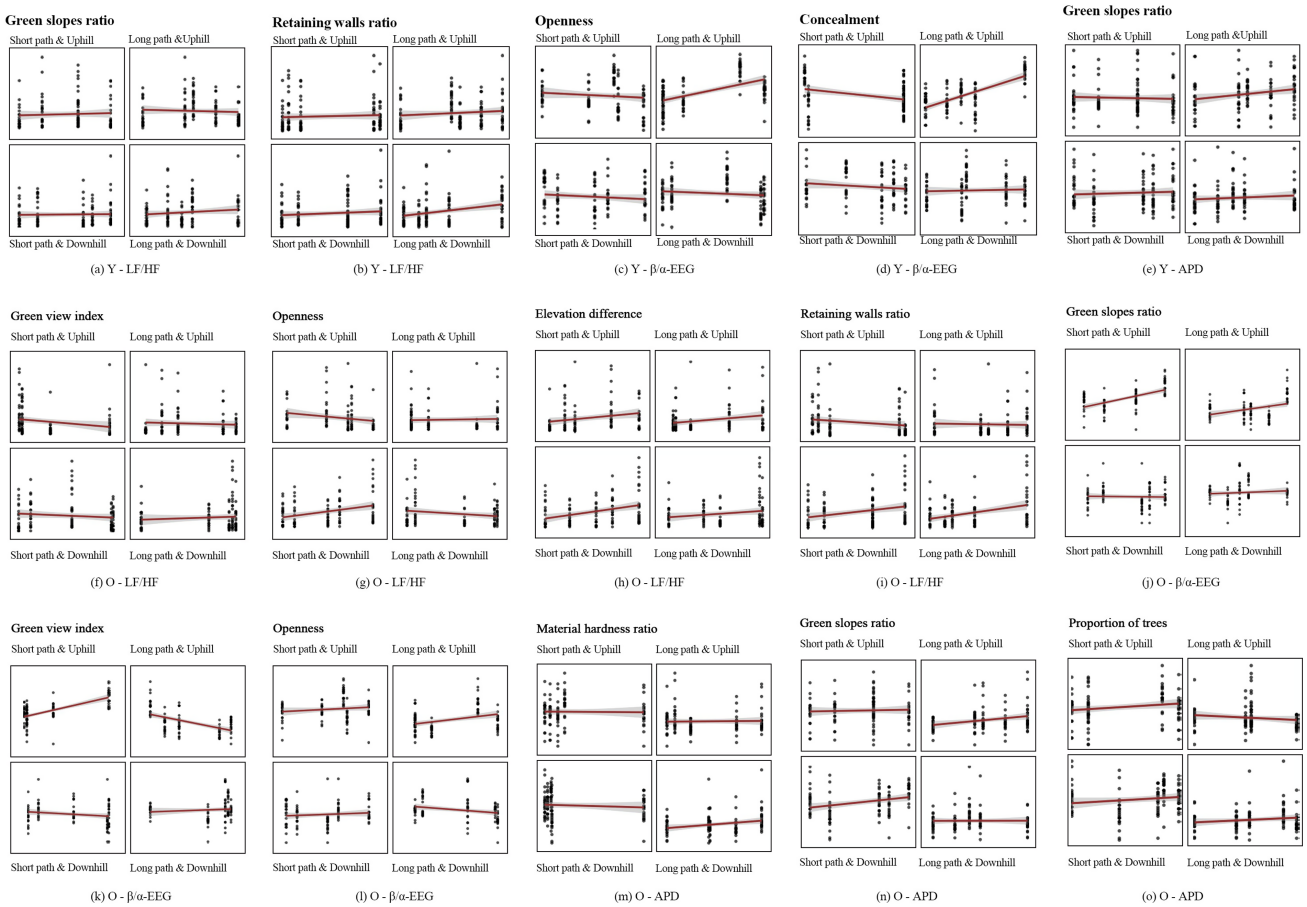
Results of the GLMM for the visual environment and physiological indicators of the youth and older adults using different paths and modes of movement.

Different spatial characteristics can have varying and significant impacts on the perceptions of young and older adult individuals (Liu et al., 2019). GLMM analysis revealed that the proportion of trees, green view index, proportion of green slopes, elevation difference, openness, and concealment all significantly affected the physiological data of young and older adult individuals (*p* < 0.05). However, elevation difference and green view index had more significant impacts on the LF/HF of older adult individuals than on young individuals. Statistical analysis of the visual environment indicators (Table 4) and the results of the GLMM analysis of the physiological data for each path (Supplementary Figures 2, 3) showed that young individuals on uphill paths had a higher degree of openness, which is associated with a gradual increase in *β*/*α*, whereas on downhill paths, the trend was the opposite (Figure 11C). For the LPU, a higher proportion of green slopes was associated with a lower LF/HF, whereas on the other three paths, the trend was the opposite (Figure 11A). For the LPD, a higher proportion of retaining walls was associated with a higher LF/HF (Figure 11B). For the SPU, greater concealment was associated with a gradual decrease in β/α (Figure 11D). For the SPD, a higher degree of openness was associated with a smaller β/α (Figure 11C). For older adult individuals, a greater elevation difference was associated with a gradual increase in LF/HF across all four paths, with a more significant impact than in young individuals (Figure 11H). Path-specific discussions indicate that during the uphill process, a higher proportion of green slopes was associated with a higher β/α (Figure 11J), whereas during the downhill process, a higher proportion of retaining walls was associated with a higher LF/HF (Figure 11I). For the LPU, a higher green view index was associated with gradual decreases in both LF/HF and β/α (Figures 11F,K). For the LPD, a higher degree of openness was associated with a smaller LF/HF (Figure 11G), and a greater material hardness ratio were associated with a larger APD (Figure 11M). For the SPU, a higher proportion of retaining walls was associated with a gradual increase in LF/HF, whereas a higher green view index was associated with a gradual decrease in LF/HF (Figures 11F,I). For the SPD, a higher degree of openness was associated with a gradual decrease in β/α (Figure 11G), and a higher proportion of green slopes was associated with a gradual increase in APD (Figure 11N). These findings indicate that the same spatial indicators in different path environments can have different physiological perception impacts on young and older adult individuals. For example, for the SPD, an increase in the green view index was associated with an increase in β/α for young individuals, whereas for older adult individuals, it was associated with a gradual decrease. For the other three paths, the trends in β/α with changes in the green view index were similar for both young and older adult individuals. For the LPU, an increase in the proportion of trees was associated with a gradual increase in the average APD for older adult individuals, whereas there was no significant change for young individuals.

**Table 4:** Statistical descriptions of the visual environment.

Path type	Parameter	Visual parameter							
	Site	Openness (%)	Green view index (%)	Concealment (%)	Material hardness ratio (%)	Retaining walls ratio (%)	Green slopes ratio (%)	Proportion of trees (%)	Elevation difference (m)
Short path, going up	(a) Start	0.42	0.45	0.75	0.82	0.25	0.29	0.19	0.00
	(b) Node1	0.36	0.35	0.95	0.55	0.24	0.18	0.30	1.44
	(c) Node2	0.35	0.66	0.74	2.59	0.12	0.39	0.19	2.4
	(d) Node3	0.29	0.34	0.98	0.96	0.10	0.29	0.28	5.83
	(e) Finish	0.18	0.35	1.00	0.66	0.09	0.11	0.17	8.47
Short path, going down	(a) Start	0.32	0.23	1.00	0.49	0.02	0.00	0.19	0.00
	(b) Node1	0.49	0.77	0.94	0.78	0.06	0.14	0.55	1.56
	(c) Node2	0.26	0.78	0.85	10.6	0.03	0.16	0.48	3.25
	(d) Node3	0.72	0.54	0.97	1.14	0.08	0.20	0.50	5.98
	(e) Finish	0.55	0.3	0.75	1.22	0.06	0.04	0.35	10.27
Long path, going up	(a) Start	0.36	0.64	0.77	2.31	0.12	0.18	0.30	0.00
	(b) Node1	0.41	0.60	0.65	1.96	0.19	0.26	0.29	0.39
	(c) Node2	0.55	0.36	0.98	1.10	0.14	0.34	0.30	2.60
	(d) Node3	0.36	0.46	0.56	0.99	0.24	0.15	0.20	8.32
	(e) Finish	0.61	0.31	0.71	1.32	0.01	0.00	0.38	13.26
Long path, going down	(a) Start	0.68	0.52	0.71	2.07	0.01	0.04	0.45	0.00
	(b) Node1	0.69	0.72	0.56	7.27	0.04	0.1	0.68	11.44
	(c) Node2	0.57	0.78	0.73	9.21	0.03	0.14	0.63	2.34
	(d) Node3	0.39	0.79	0.90	5.22	0.14	0.17	0.50	5.59
	(e) Finish	0.37	0.80	0.98	5.31	0.03	0.30	0.29	0.13

**Figure 12:**
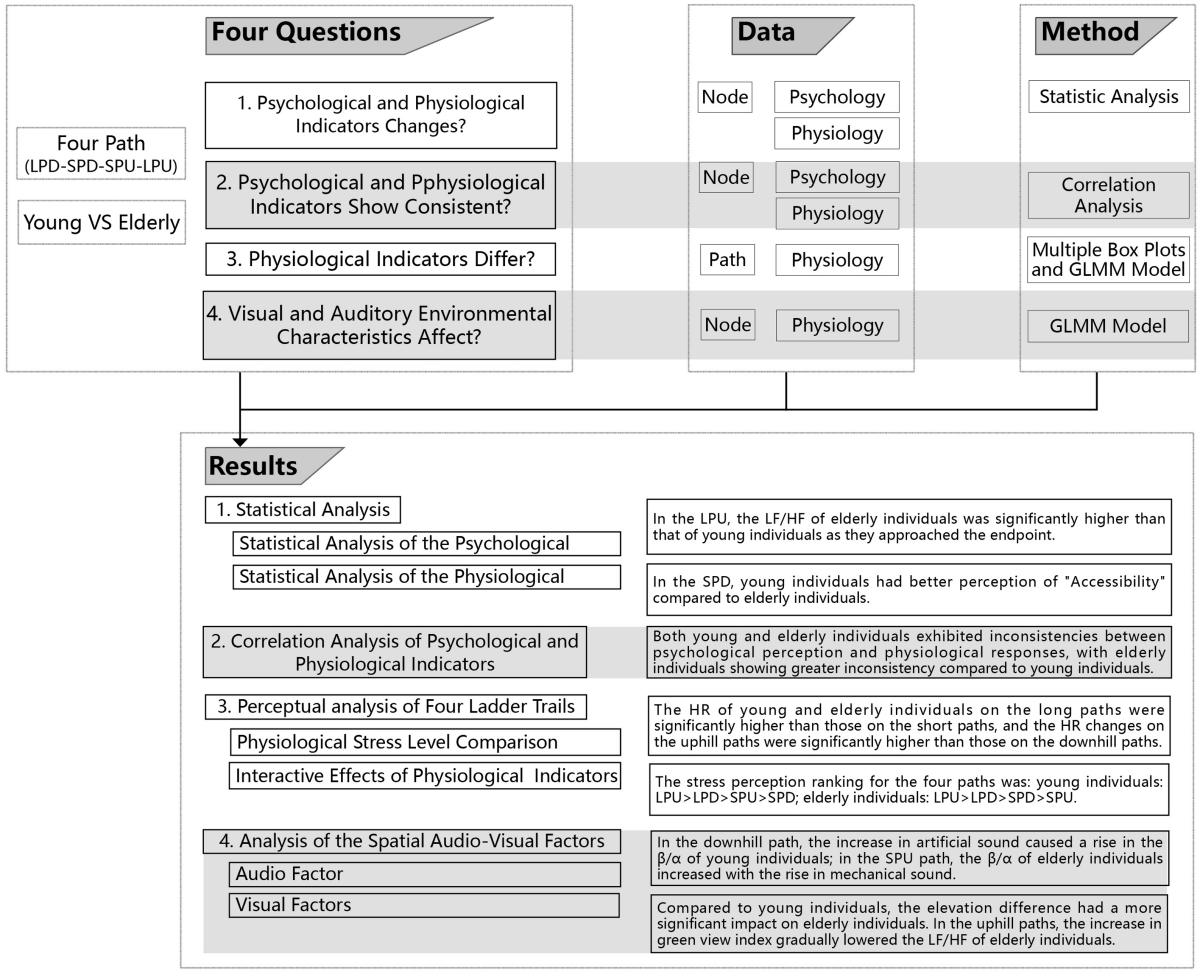
Results framework.

The framework of the results is shown in Figure 12. Similar to the proposed hypotheses, the psychological indicators of young and older adult individuals differed across the four path types, and physiological indicators changed at various nodes as the experiment progresses; compared to young individuals, older adult individuals exhibited a greater inconsistency between psychological perception and physiological responses; both young and older adult individuals experienced greater stress when walking on LPU than when walking on other three path types. Additionally, elevation differences, openness, and natural elements were the primary factors influencing the perception of both young and older adult individuals. Further analysis will be conducted based on these findings.

## 4 Discussion

### 4.1 Changes and differences in psychological and physiological indicators

Descriptive statistics of the collected psychological and physiological data indicate that in terms of the psychological perception results, young and older adult individuals showed distinct differences in the evaluation of certain indicators across the four paths. For example, older adult individuals gave more negative evaluations for the “Anxiety and Calmness” and “Monotony” indicators than young individuals. In contrast, young individuals rated “Accessibility” and “Naturalness” more favourably than older adult individuals. Young individuals tend to have a stronger goal-oriented approach and greater vitality, along with a higher threshold for perceiving fatigue (Mcardle et al., 2015), which explains their higher evaluation of the “Accessibility” of the path. This analysis suggests that, in mountain park stairway spaces, older adult individuals require more resting spaces, whereas young individuals benefit from enriched audiovisual environments and attractive spaces. Resting and activity spaces in mountain city parks are largely constrained by the terrain; however, diverse soundscapes can be created by utilising terrain features and integrating acoustic environments within the park. Stairway spaces with varied elevation changes and diverse vegetation layers should be established, while reserving adequate resting areas to optimise the perceptual experience for different demographic groups in stairway spaces. Such design strategies have been proposed and validated in the studies of Yao et al. (2022) and Dai et al. (2022). Regarding the trend of physiological indicators, young individuals exhibited greater adaptability to the vibrant public square and audiovisual environments at the end of the LPU path in the mountain park stairway space, and physiological indicators such as LF/HF and APD showed significant variation as they approached the endpoint (Figures 3, 4). Zhang Y. et al. (2022) and Zhang Z. et al. (2022) found that appropriately adding adventurous and vibrant nodes in parks can enhance the physical and mental restorative benefits for young individuals. As expected, older adult individuals exhibited more stable physiological responses in the stairway space.

### 4.2 Consistency between psychological perception and physiological responses

An analysis of the correlation between the psychological perceptions and physiological responses of young and older adult individuals revealed that, on the SPU, older adults’ evaluation of the “Diversity” indicator showed a significant positive correlation with their RESP. On the short path, older adults rated the landscape’s diversity positively; however, their respiratory rate gradually increased, and their perception of stress also heightened. While previous studies have shown that diverse landscape environments promote physiological relaxation (Jeon et al., 2023), the inconsistency observed in this study can be explained by the research of Chang et al. (2020) and Copeland (2013), which suggests that ageing leads to a slowdown in neural signal transmission efficiency, resulting in a misalignment between psychological perception and physiological responses. Additionally, owing to older adults’ behavioural preferences, they tend to spend more time in environments such as parks and green spaces, leading to an adaptation to similar environments. Therefore, the impact of a diverse landscape on their perception is relatively minimal, while the increased physical load during the uphill process has a more significant effect on their physiological responses. Moreover, the consistency between psychological perception and physiological responses exhibited by young and older adult individuals across the four paths corroborates some existing research conclusions. For instance, the psychological perception indicator of “Functionality” showed a negative correlation with the older adults’ LF/HF ratio during the uphill process, whereas the opposite trend was observed for the young individuals. This result aligns with the findings of Zhang Y. et al. (2022) and Zhang Z. et al. (2022), which suggested that older adult individuals prefer environments that provide good shelter and a sense of security. This preference is consistent with the present study’s observation that the higher older adults’ perception of “Openness,” the higher their *β*/*α* and the greater their perceived stress. These conclusions preliminarily indicate that there is a certain degree of inconsistency between the psychological perceptions and physiological responses of young and older adult individuals across the four paths, with older adults showing more significant inconsistency than the young. In the design of stairway spaces in mountain city parks, it is crucial to fully consider older adults’ physical capabilities and behavioural preferences. Additionally, based on the functional needs of different age groups, optimising facility configurations to meet the diverse experiential needs of both young and older adult individuals will enhance the park’s accessibility and universality.

### 4.3 Varying effects of populations, paths, and modes of movement

Multiple box plots (Figure 6) and the GLMM (Table 3) showed that the physiological perception varies under the influence of different paths, movement modes, age groups, and their interaction effects. The perception of stress was significantly higher on long paths than on short paths and significantly greater during uphill than downhill movements. Additionally, young individuals exhibited significantly higher stress perception than older adult individuals. In long paths, landscape variation was minimal, with a higher degree of concealment, resulting in a certain monotony in visual perception. Moreover, the substantial elevation difference made these paths less attractive to young individuals. In contrast, short paths were equipped with fitness equipment, leisure corridors, and other infrastructure, featuring a visually enriched environment. Furthermore, the endpoints of these short paths served as social activity spaces. Older adult individuals tended to use basic infrastructure for low-intensity exercise and perceived that fitness and recreational facilities in the park contributed to health improvement, providing spaces for social interaction and relaxation, which helped alleviate their stress perception (Chow, 2013). Taczanowska et al. (2024) found that young individuals have higher demands for visual stimulation in the environment, preferring diversity and novelty and emphasising social interaction and personalised experiences. Monotonous and highly concealed environments have been shown to elicit negative emotions in young individuals (Khalifa et al., 2024). Dai et al. (2022) further supported this conclusion, demonstrating that young individuals are more inclined toward park environments featuring varied topography and stimulating activities to fulfil their needs for challenge and adventure. In addition, the results of the GLMM analysis showed that second- and third-order interactions between path, movement mode, and age group significantly affected the physiological responses of participants. Third-order interactions significantly affected APD in young and older adult individuals. These results indicate that participants’ perceptions are influenced by visual and auditory environments, behaviour, and other factors. For example, in the LPD, although the visual environment does not change substantially, the path features a relatively rich variety of sounds and multiple turns, attracting the attention of both young and older adult individuals. This confirms, to some extent, that the auditory environment influences users’ perception of the visual environment and vice versa (Banks et al., 2012; Southworth, 1969). For older adult individuals, the auditory environment has a greater impact on their perception than the visual environment (Ruotolo et al., 2024). Therefore, diverse soundscapes can be created or maintained by leveraging the openness that inherently characterises mountain parks. By comprehensively considering both visual and auditory environments, perception can be improved through multisensory integration mechanisms.

### 4.4 Impacts of mountain environmental factors on the physiological perceptions of young and older adult individuals

The GLMM effectively revealed the influence of auditory and spatial environmental factors on participants’ psychological and physiological perceptions. The significant elevation difference in mountain city parks makes stairway spaces important for organising traffic and providing rest and recreational opportunities. Additionally, “Elevation difference” becomes a critical factor influencing users’ psychological and physiological perceptions (Jia et al., 2020). As expected, the impact of elevation difference on older adults’ LF/HF ratio was greater than that on the young. Across the four paths, an increase in elevation was accompanied by a rise in older adults’ LF/HF, which was linked to increased physical load and perceived stress. However, on the uphill paths, the gradually increasing “Proportion of Green Slopes” and “Concealment” resulted in a gradual reduction in older adults’ LF/HF, which alleviated their stress to some extent. Mechanical noise in the SPU path caused a noticeable increase in *β*/*α* for both young and older adult participants, as this path was located near a major urban road with a significant elevation difference. Zhang J. et al. (2024) and Zhang R. et al. (2024) found that mechanical noise and other types of noise induce significant negative emotions and increase cognitive load in young individuals. Mexi et al. (2023) reported that uphill movement increases the risk of musculoskeletal injuries and falls, whereas downhill movement demonstrates higher gait stability. Older adult individuals are generally more sensitive to low-frequency mechanical noises, such as traffic noise, than young individuals (Berglund et al., 1996; Kurakata et al., 2008). Furthermore, the physical decline associated with ageing leads to an increased physical load during uphill movement, exacerbating the generation of negative emotions. Therefore, the increased mechanical sounds in the SPU significantly heightened stress perception in both young and older adult individuals. In the LPU, an increase in the green view index and natural sounds resulted in a gradual decrease in β/α, whereas in the SPU, the opposite trend was observed. This may be attributed to the fact that the short path, serving as a primary vertical circulation route, features a relatively monotonous landscape configuration. In such an environment, participants were required to maintain a high level of spatial attention. Notably, β/α peaked between Nodes 2 and 3. Although the green view index increased, the proportion of retaining walls also rose accordingly, which partially obstructed visibility, heightened participants’ attentional demands, and introduced a certain degree of psychological stress, thereby influencing their physiological responses. The long path, primarily used for sightseeing, has rest platforms along the stairway path, with the green view index gradually increasing after the starting point. According to White et al. (2021), frequent exposure to green and blue spaces can significantly enhance mental health, with blue spaces being more effective at promoting relaxed and pleasant emotions. This helps explain why natural green spaces with a high proportion of natural elements can foster positive emotions during a relaxing sightseeing experience. It also suggests that in the renovation and design of mountain park stairway spaces, appropriately incorporating and increasing the proportion of blue spaces, using elevation differences to create varied artificial water features, and enhancing natural water sounds in the auditory environment can effectively improve the spatial experiences of participants (Mexi et al., 2023). However, it is worth noting that in the case of SPD, an increase in the green view index leads to a higher β/α among young participants, indicating a stronger sense of curiosity toward the environment. The presence of greenery tends to stimulate a greater desire for active perception and interaction with the surroundings, resulting in heightened cognitive arousal and increased attention, which in turn raises the β/α.

### 4.5 Application

The significance and potential applications of this study are multifaceted. First, the results provide theoretical support to help alleviate stress perceptions and improve environmental quality in the stairway spaces of mountain city parks. Stairway spaces in mountain city parks are crucial for defining three-dimensional ecological public spaces, organising vertical transportation, and guiding users to perceive the park environment. Consequently, improving the perception of stairway spaces can effectively enhance users’ overall satisfaction with mountain city parks. Second, this study focused on the perceptual differences of users in stairway spaces within mountain environments and explored the impact of the spatial factors that represent mountain characteristics on different groups. The results indicate that to alleviate users’ stress perception in stairway spaces, designers should consider the landscape preferences, physical functions, and behavioural characteristics of different groups to formulate specific optimisation and design strategies. Third, the results can be effectively integrated into existing renewal and renovation processes for mountain city parks. For example, mountain spatial indicators closely associated with participants’ perceptions, as shown in this study, can serve as reference indicators for subsequent evaluations of mountain city parks. In regeneration designs, the actual needs of various groups can be combined to improve the sound environment in stairway spaces, create suitable soundscapes, purposefully construct artificial landscapes, and adjust the green view index, openness, and material hardness ratio to maximise the landscape benefits of mountain city parks.

### 4.6 Limitations and future work

This study has a few limitations. First, although older adult participants were recruited from the local residents near the park, some of the young participants were university students between 18 and 22 years of age, and consequently, they may not fully represent the perceptions of all young park users. Additionally, due to experimental constraints, the overall sample size was limited. Second, the experiment selected a representative mountain park stairway space in a mountain city, which may not represent the characteristics of all mountain city parks and stairway spaces. Future research should compare it with other types of stairway spaces. Third, the experiment was conducted in a real environment, where some external factors could not be fully controlled. Consequently, participants’ perceptual responses may have been influenced by various factors, including visual, auditory, and olfactory stimuli. Additionally, the experiment did not consider the participants’ physical activity levels, which may have impacted their perception of stress along the paths. Fourth, due to terrain constraints, the sequence of path traversal was fixed, which may have introduced potential order effects and slightly affected the accuracy of the results. Fifth, for the data analysis, multidimensional perceptual data, including complex data types and large data volumes, were obtained. However, the analysis methods used in the experiment could only reveal the preliminary relationships between space and perception. In future studies, it is essential to include a more diverse range of user types, such as individuals with different backgrounds and genders, and further expand the sample size to draw more generalizable conclusions. Second, a comparative analysis of mountain city parks with varying environmental characteristics and different types of stairway spaces could enhance the generalisability of the findings. Third, considering the participants’ physical activity level as a factor would help refine the experimental design. Additionally, integrating additional sensory dimensions such as olfaction and touch with vision and hearing in natural environments would provide a more comprehensive validation of the research conclusions. Fourth, future studies could consider adopting randomized path sequences in experimental design to eliminate potential order effects caused by fixed traversal sequences, thereby improving the accuracy of the data. Fifth, machine learning techniques, including artificial neural networks, should be further employed to explore the potential latent influences between spatial environments and human perception.

## 5 Conclusion

This study focused on mountain city parks, where stairway spaces function as important linear connections to platforms of different heights and influence users’ overall perceptions of a park. This study considered the age group, environment, and movement mode and used a GLMM to quantitatively analyse the “psychological-physiological” aspects within mountain park environments by exploring the perceptual differences between young and older adult individuals on stairway paths of different lengths and movement directions. The study used eight experimental control groups (48 valid samples in total) to statistically describe perception questionnaire data and objective physiological data obtained from young and older adult individuals during the experiments. The consistency and differences in psychological-physiological data across the four paths and the impact of spatial factors on the physiological data were analysed and the following conclusions were drawn:

1. There are differences in the psychological and physiological perceptions of young and older adult individuals, with greater psychological differences observed in uphill paths. The uphill movement mode caused an overall upward trend in RESP, LF/HF, and *β*/*α* for both groups.
2. The psychological and physiological perceptions of young and older adult individuals were inconsistent across the four paths; however, young individuals showed higher consistency than older adult individuals and exhibited more positive psychological responses.
3. Significant differences were observed in the physiological data of young and older adult individuals across the four pathways. The stress perception was greater on long paths than on short paths. However, the sense of insecurity and knee burden caused by elevation differences lead to greater stress perception in older adult individuals on the SPD than on the uphill path.
4. Spatial factors such as elevation difference, proportion of green slopes, and openness, which are mountain characteristics, are major factors influencing physiological changes in young and older adult individuals; however, their impacts on the different groups vary.

Previous research has revealed that natural elements significantly reduce user stress perception. The current study validated this notion because the results demonstrate that natural elements, such as the green view index and concealment, can alleviate users’ emotions in most cases. However, under specific conditions, owing to differences in the physical functions of young and older adult individuals, gait differences in uphill and downhill movements, and the comprehensive impact of other visual and auditory elements, an increase in the green view index can also lead to increased stress perception. For example, for young SPD users, the increase in the green view index and concealment makes the landscape monotonous; consequently, it does not attract their interest, and the downhill posture exerts pressure on a person’s knees. The combined effects of multiple factors can lead to negative emotions in young individuals on certain paths, whereas the reactions of older adult individuals are less pronounced. Assessments based on the sound environment were consistent with previous research. Natural sounds can alleviate stress perceptions in both young and older adult individuals, whereas artificial and mechanical sounds cause more significant negative emotions in young individuals. The environment and movement modes also influenced young and older adult individuals’ perceptions. In addition, in the same environment, the multidimensional perceptions of the young and older adult groups may differ owing to perceptual preference factors. Meanwhile, the study identified differences in perceptual change trends among different user groups through the consistency between psychological perception and physiological responses, and the establishment of the GLMM model further identified key spatial elements that influence different user groups, providing crucial theoretical support for the optimised design of stairway spaces in mountain parks. Future research should consider the perceptual differences of more diverse populations and the intrinsic impacts of other sensory environmental characteristics on embodied perception to support the development of mountain city parks that are friendly to all ages.

## Data Availability

The raw data supporting the conclusions of this article will be made available by the authors, without undue reservation.
