# Supplementary material for: Multidimensional on-site perception study of stairway spaces in mountain city parks among young and older adult people: a case study of Pipa Mountain Park, Chongqing, China
Source: Front Psychol. 2025 Aug 7;16:1620884. doi: 10.3389/fpsyg.2025.1620884 (PMC12371939; doi:10.3389/fpsyg.2025.1620884)
Supplement: Supplementary file 1 [file Supplementary_file_1.docx]

Supplementary Material

# Supplementary Figures and Tables

**Supplementary Table 1 GLMM results**

|  | **Effect** | **Estimate** | **Std.** | **t value** | **Sig.** |
| --- | --- | --- | --- | --- | --- |
| Heart rate (HR) | Path | 0.23 | 0.3 | 0.77 |  |
|  | Motion | 0.21 | 0.3 | 0.71 |  |
|  | People | −0.17 | 0.3 | −0.55 |  |
|  | Path️*Motion | −0.19 | 0.19 | −1.02 |  |
|  | Path*People | 0.02 | 0.19 | 0.11 |  |
|  | Motion*People | −0.08 | 0.19 | −0.42 |  |
|  | Path*People*Motion | 0.04 | 0.12 | 0.36 |  |
| Respiratory rate (RESP) | Path | 0.41 | 0.26 | 1.56 | * |
|  | Motion | 0.49 | 0.26 | 1.88 | * |
|  | People | 0.28 | 0.27 | 1.04 |  |
|  | Path*Motion | −0.31 | 0.17 | −1.88 | * |
|  | Path*People | −0.19 | 0.17 | −1.13 |  |
|  | Motion*People | −0.26 | 0.17 | −1.55 |  |
|  | Path*People*Motion | 0.16 | 0.11 | 1.5 |  |
| Low-Frequency/High-Frequency Ratio (LF/HF) | Path | −0.33 | 0.26 | −1.25 |  |
|  | Motion | −0.14 | 0.26 | −0.52 |  |
|  | People | −0.34 | 0.27 | −1.29 |  |
|  | Path*Motion | 0.13 | 0.17 | 0.82 |  |
|  | Path*People | 0.25 | 0.17 | 1.51 | * |
|  | Motion*People | 0.14 | 0.17 | 0.82 |  |
|  | Path*People*Motion | −0.11 | 0.11 | −1.03 |  |
| α-EEG | Path | 0.24 | 0.31 | 0.78 |  |
|  | Motion | 0.24 | 0.31 | 0.76 |  |
|  | People | 0.33 | 0.32 | 1.03 |  |
|  | Path*Motion | −0.10 | 0.20 | −0.51 |  |
|  | Path*People | −0.16 | 0.20 | −0.80 |  |
|  | Motion*People | −0.15 | 0.20 | −0.73 |  |
|  | Path*People*Motion | 0.08 | 0.13 | 0.63 |  |
| β-EEG | Path | 0.03 | 0.30 | 0.10 |  |
|  | Motion | −0.01 | 0.30 | −0.04 |  |
|  | People | 0.25 | 0.31 | 0.80 |  |
|  | Path*Motion | 0.11 | 0.19 | 0.57 |  |
|  | Path*People | −0.03 | 0.19 | −0.17 |  |
|  | Motion*People | 0.00 | 0.19 | 0.02 |  |
|  | Path*People*Motion | −0.06 | 0.12 | −0.46 |  |
| β/α-EEG | Path | −0.46 | 0.31 | −1.51 | * |
|  | Motion | −0.34 | 0.31 | −1.10 |  |
|  | People | −0.66 | 0.32 | −2.13 | * |
|  | Path*Motion | 0.14 | 0.19 | 0.72 |  |
|  | Path*People | 0.29 | 0.20 | 1.50 | ** |
|  | Motion*People | 0.24 | 0.20 | 1.23 |  |
|  | Path*People*Motion | −0.10 | 0.12 | −0.77 |  |
| Fixation frequency (FF) | Path | 0.10 | 0.20 | 0.45 |  |
|  | Motion | 0.12 | 0.20 | 0.58 |  |
|  | People | 0.15 | 0.21 | 0.73 |  |
|  | Path*Motion | −0.11 | 0.13 | −0.88 |  |
|  | Path*People | −0.15 | 0.13 | −1.12 |  |
|  | Motion*People | −0.19 | 0.13 | −1.42 | * |
|  | Path*People*Motion | 0.19 | 0.08 | 2.26 | * |
| Average pupil diameter (APD) | Path | 0.62 | 0.24 | 2.59 | * |
|  | Motion | 0.44 | 0.24 | 1.83 |  |
|  | People | 0.40 | 0.24 | 1.64 |  |
|  | Path*Motion | −0.41 | 0.15 | −2.70 | * |
|  | Path*People | −0.43 | 0.16 | −2.78 | * |
|  | Motion*People | −0.35 | 0.16 | −2.29 | * |
|  | Path*People*Motion | 0.33 | 0.10 | 3.40 | * |
| Saccade frequency (SF) | Path | 0.34 | 0.26 | 1.31 |  |
|  | Motion | 0.38 | 0.26 | 1.48 | * |
|  | People | 0.29 | 0.26 | 1.11 |  |
|  | Path*Motion | −0.26 | 0.16 | −1.59 | * |
|  | Path*People | −0.08 | 0.17 | −0.46 |  |
|  | Motion*People | −0.07 | 0.17 | −0.40 |  |
|  | Path*People*Motion | 0.00 | 0.10 | 0.00 |  |
|  | | | | | |

Note: ‘*’ indicates a significant correlation at the 0.05 level, and ‘**’ indicates a significant correlation at the 0.01 level.


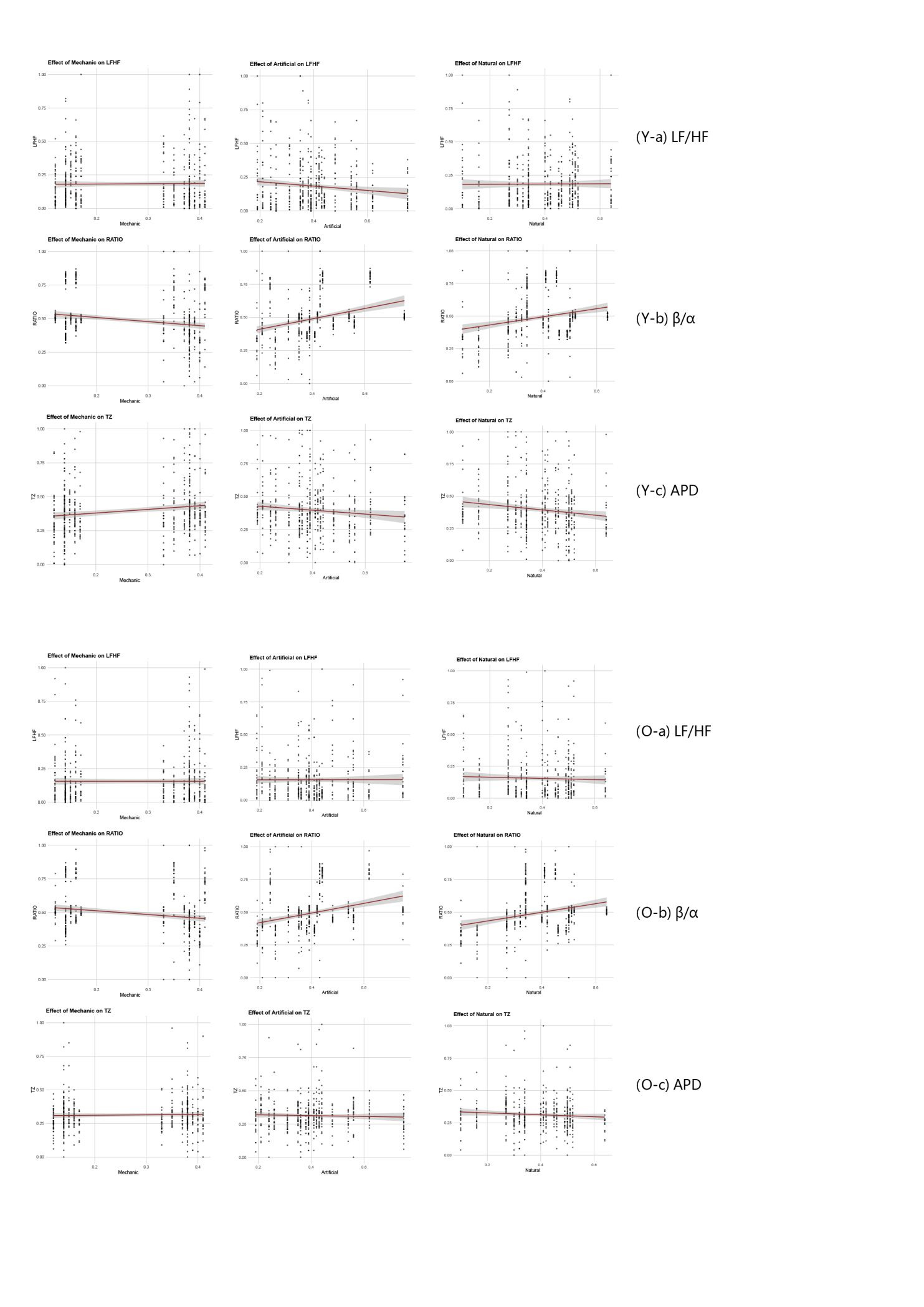


**Supplementary Figure 1.** GLMM results showing the impact of the acoustic environment on physiological indicators in young and elderly individuals.


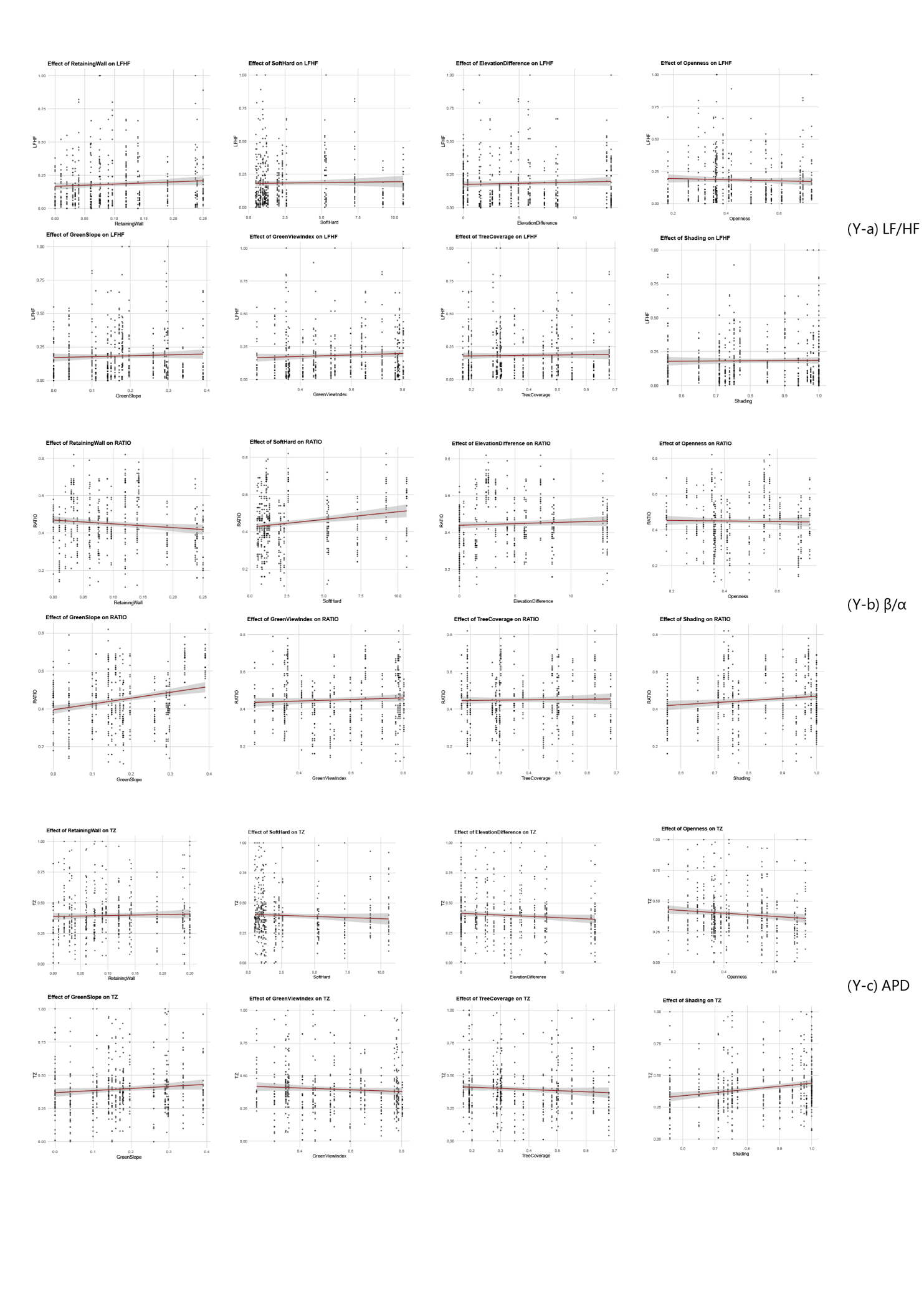


**Supplementary Figure 2.** GLMM results showing the impact of the visual environment on physiological indicators in young individual.


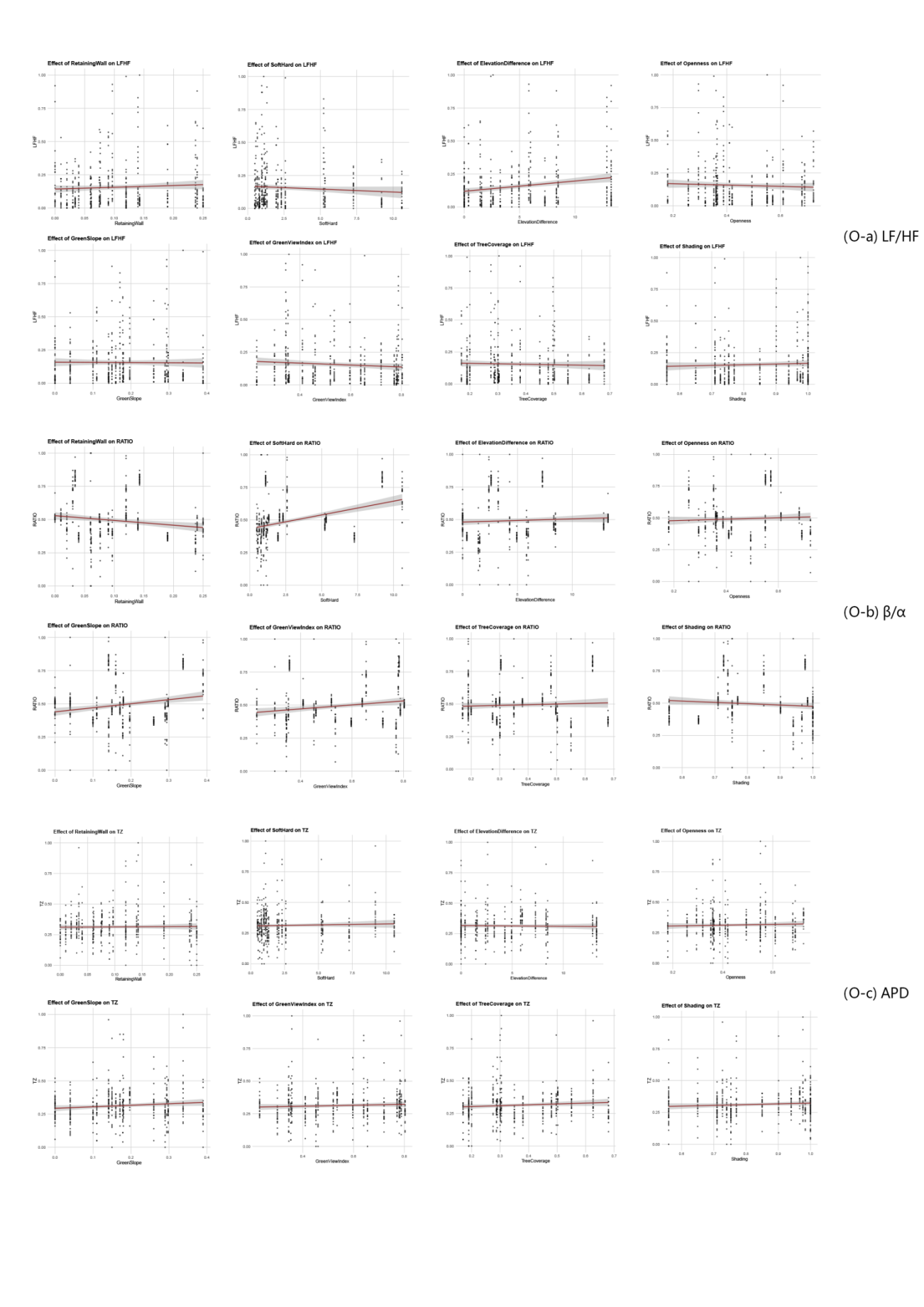


**Supplementary Figure 3.** GLMM results showing the impact of the visual environment on physiological indicators in elderly individual.


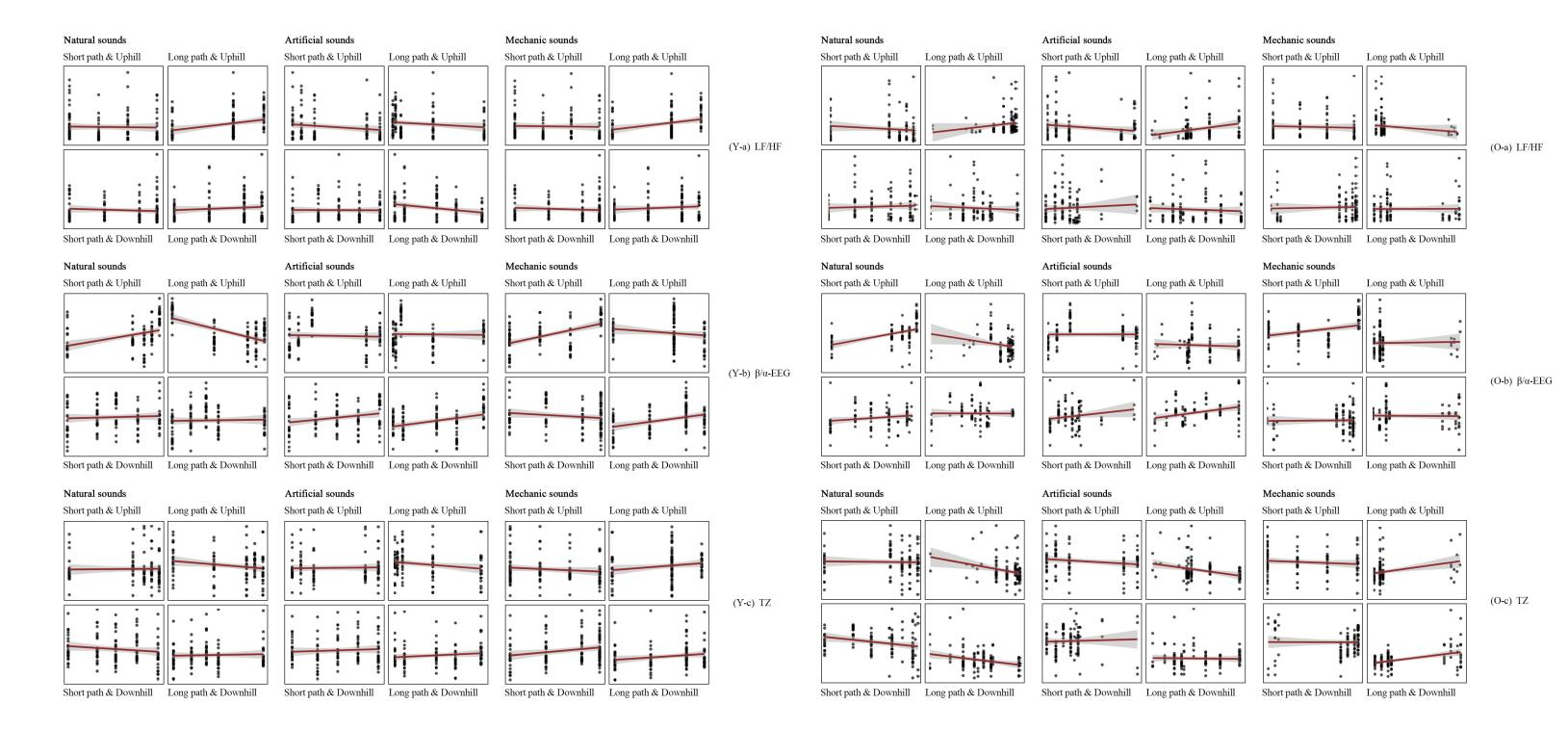


**Supplementary Figure 4.** Results of the GLMM for the acoustic environment and physiological indicators of different groups, paths, and modes of movement.


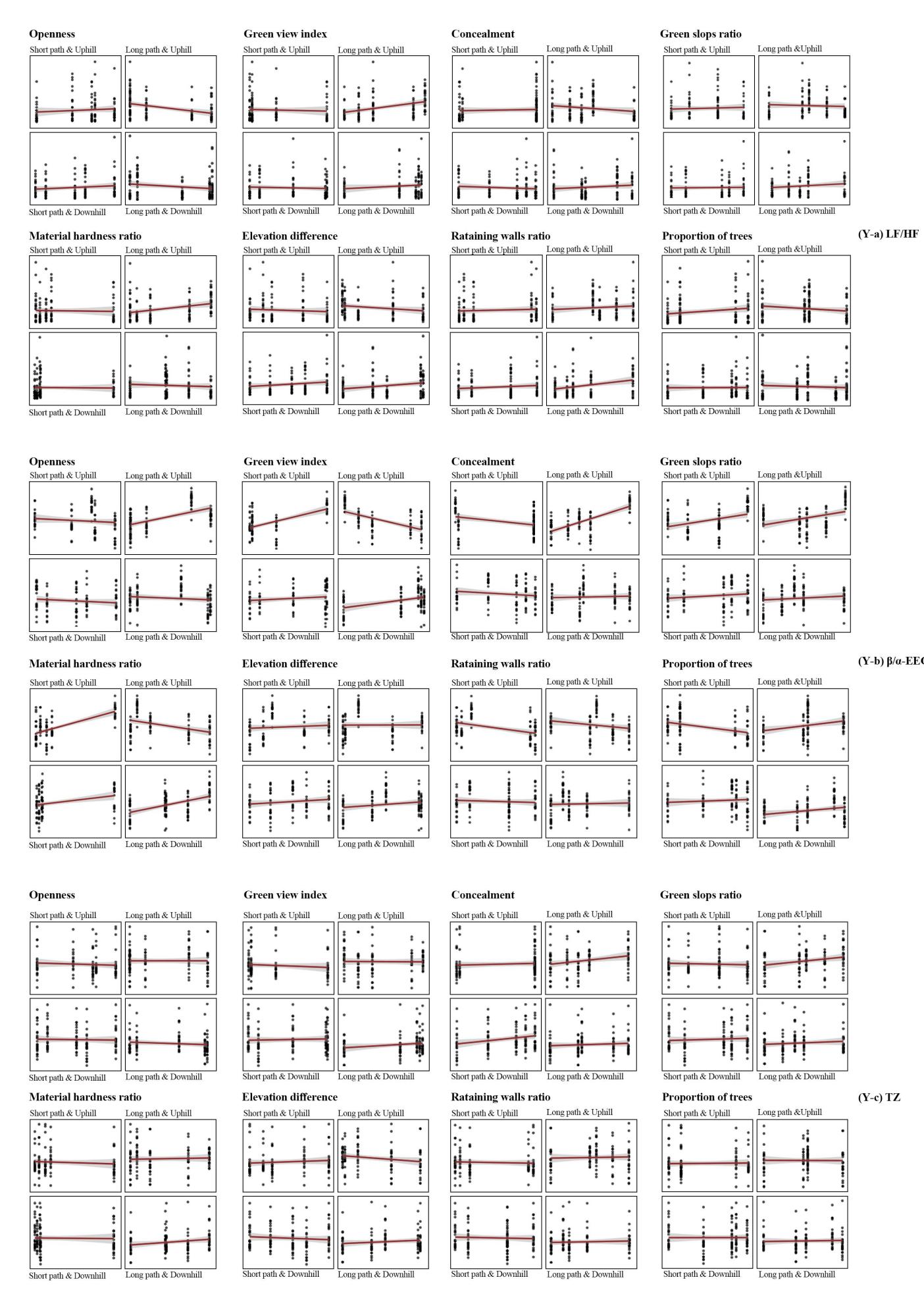


**Supplementary Figure 5.** Results of the GLMM for spatial and physiological indicators of youth using different paths and modes of movement.


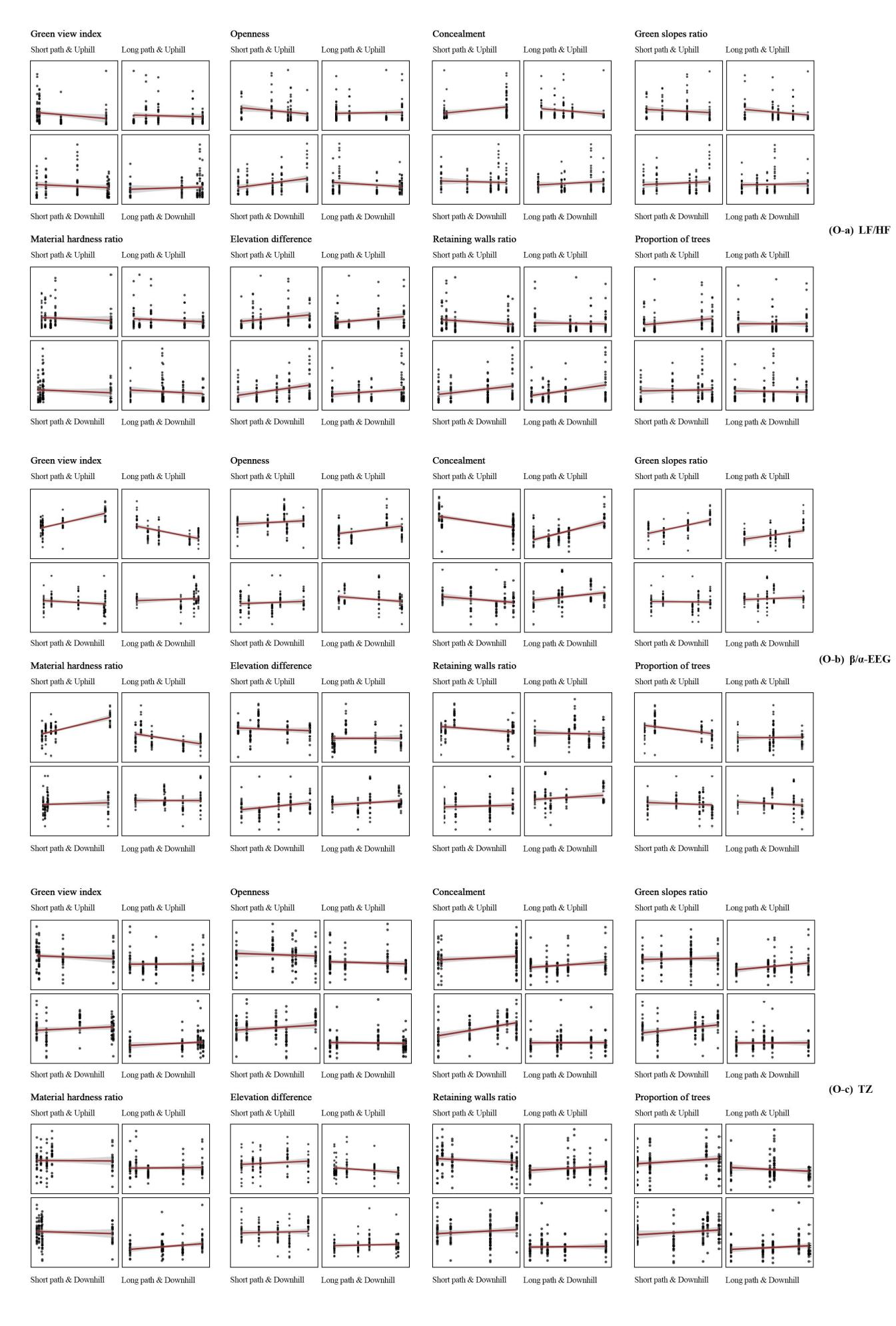


**Supplementary Figure 6.** Results of the GLMM for the visual environment and physiological indicators of the elderly using different paths and modes of movement.
